# Crosstalk between leukocytes triggers differential immune responses against *Salmonella enterica* serovars Typhi and Paratyphi

**DOI:** 10.1371/journal.pntd.0007650

**Published:** 2019-08-14

**Authors:** Rosangela Salerno-Goncalves, Darpan Kayastha, Alessio Fasano, Myron M. Levine, Marcelo B. Sztein

**Affiliations:** 1 Center for Vaccine Development and Global Health, Department of Pediatrics, University of Maryland School of Medicine, Baltimore, MD, United States of America; 2 Mucosal Immunology and Biology Research Center, Massachusetts General Hospital for Children, Boston, MA, United States of America; Mohammed Bin Rashid University of Medicine and Health Sciences, UNITED ARAB EMIRATES

## Abstract

Enteric fevers, caused by the *Salmonella enterica* serovars Typhi (ST), Paratyphi A (PA) and Paratyphi B (PB), are life-threatening illnesses exhibiting very similar clinical symptoms but with distinct epidemiologies, geographical distributions and susceptibilities to antimicrobial treatment. Nevertheless, the mechanisms by which the host recognizes pathogens with high levels of homology, such as these bacterial serovars, remain poorly understood. Using a three-dimensional organotypic model of the human intestinal mucosa and PA, PB, and ST, we observed significant differences in the secretion patterns of pro-inflammatory cytokines and chemokines elicited by these serovars. These cytokines/chemokines were likely to be co-regulated and influenced the function of epithelial cells, such as the production of IL-8. We also found differing levels of polymorphonuclear leukocyte (PMN) migration among various infection conditions that either included or excluded lymphocytes and macrophages (Mϕ), strongly suggesting feedback mechanisms among these cells. Blocking experiments showed that IL-1β, IL-6, IL-8, TNF-α and CCL3 cytokines were involved in the differential regulation of migration patterns. We conclude that the crosstalk among the lymphocytes, Mϕ, PMN and epithelial cells is cytokine/chemokine-dependent and bacterial-serotype specific, and plays a pivotal role in orchestrating the functional efficiency of the innate cells and migratory characteristics of the leukocytes.

## Author summary

Enteric fevers are acute illnesses caused mainly by the *Salmonella enterica* serovars Typhi (ST), Paratyphi A (PA) and Paratyphi B (PB). The incidence of enteric fevers is very low in industrialized countries, with most infections occurring in military personnel and individuals traveling to endemic areas. Nevertheless, worldwide, more than 20.6 million cases of enteric fever occur annually in low- and middle-income countries and are associated with approximately 129,000–223,000 deaths. ST, PA, and PB exhibit very similar clinical symptoms but distinct epidemiologies, geographical distributions, and susceptibilities to antimicrobial treatment. However, the mechanisms by which the host recognizes pathogens with high levels of homology, such as these bacterial serovars, remain poorly understood. Using an *in vitro* model of the human intestinal mucosa, we found that the crosstalk among leukocytes—lymphocytes, Mϕ, and PMN- and epithelial cells is cytokine/chemokine-dependent, and bacterial-serotype specific and plays a pivotal role in orchestrating the functional efficiency of the innate cells and migratory characteristics of the leukocytes.

## Introduction

Typhoid and paratyphoid fevers are known as enteric fevers and are caused by the intracellular Gram-negative bacteria *Salmonella enterica* serovars Typhi (ST), Paratyphi A (PA) and Paratyphi B (PB)[1–4]. Enteric fevers are rare in industrialized countries with most infections occurring in military personnel and travelers to endemic areas. The Centers for Disease Control and Prevention (CDC), in the United States, report approximately 400 laboratory-confirmed cases per year [5]. Nevertheless, ST and PA infections are a significant public health problem in the developing world [4, 6–10]. Each year, 11.9–20.6 million new cases of typhoid fever occur in low- and middle-income countries and are associated with approximately 129,000–223,000 deaths [11–14]. These deaths happen mostly in Asia in children under five years of age [15]. Additionally, the emergence of multidrug-resistant strains of ST and PA has complicated the antimicrobial treatment of enteric fever and asymptomatic carriers [16–19]. To address this public health problem, there is an increased emphasis on sanitation measures, such as water and sewage treatment, and vaccination to fight these infections [4].

Interestingly, typhoid and paratyphoid fevers exhibit very similar clinical features [2, 4, 20] but distinct epidemiologies, geographical distributions, and susceptibilities to antimicrobial treatment [21–23]. Although vaccines for PA and PB are not readily available, the existence of microbiological similarities among PA, PB, and ST, and the cross-reactivity elicited by the Ty21a typhoid vaccine against PA [24–27] and PB [27–29] support the feasibility of developing a Paratyphoid vaccine [30]. For example, the Center for Vaccine Development at the University of Maryland, Baltimore is currently evaluating a mucosally administered attenuated PA vaccine candidate [30, 31]. In humans, the only reservoir for these infections, the disease spreads by the fecal-oral route via contaminated food and water [4, 10]. ST, PA, and PB adhere to and invade the distal ileum epithelium and, subsequently, disseminate to cause enteric fevers. Intestinal epithelium and immune cells play a pivotal role in sensing and directing immune responses to maintain homeostasis [32, 33]. The crosstalk among these cells is critical in regulating intestinal innate and subsequent adaptive immune responses against bacterial pathogens [32, 33]. Thus, vaccination strategies to prevent enteric fevers, as well as therapeutic interventions to treat *Salmonella* infection, require detailed information on the early events of host responses to *Salmonella*.

Yet, the role of crosstalk among innate cells at the site of infection, and how this crosstalk influences the host response to serovars that share a high degree of homology remains unexplored. Here, using a multicellular three-dimensional (3-D) organotypic model of the human intestinal mucosa composed of multiple cell types, which was developed and characterized by our group [34–39], and PA, PB, and ST, we observed significant differences in the secretion patterns of pro-inflammatory cytokines and chemokines elicited by these serovars. These cytokines/chemokines were likely to be co-regulated and influenced the function of epithelial cells, such as the production of IL-8. We also found differing levels of polymorphonuclear leukocyte (PMN) migration among the infection conditions that either included or excluded lymphocytes and macrophages (Mϕ), strongly suggesting feedback mechanisms among these cells. Blocking experiments showed that IL-1β, IL-6, IL-8, TNF-α and CCL3 cytokines were involved in these migratory differences. Thus, the crosstalk among the lymphocytes, Mϕ, PMN and epithelial cells was cytokine-dependent and bacterial-serotype specific, and likely to play a pivotal role in orchestrating the functional efficiency of innate cells and migratory characteristics of the leukocytes.

## Results

### Differential patterns of cytokine secretion after exposure to ST, PA, and PB

**Fig 1:**
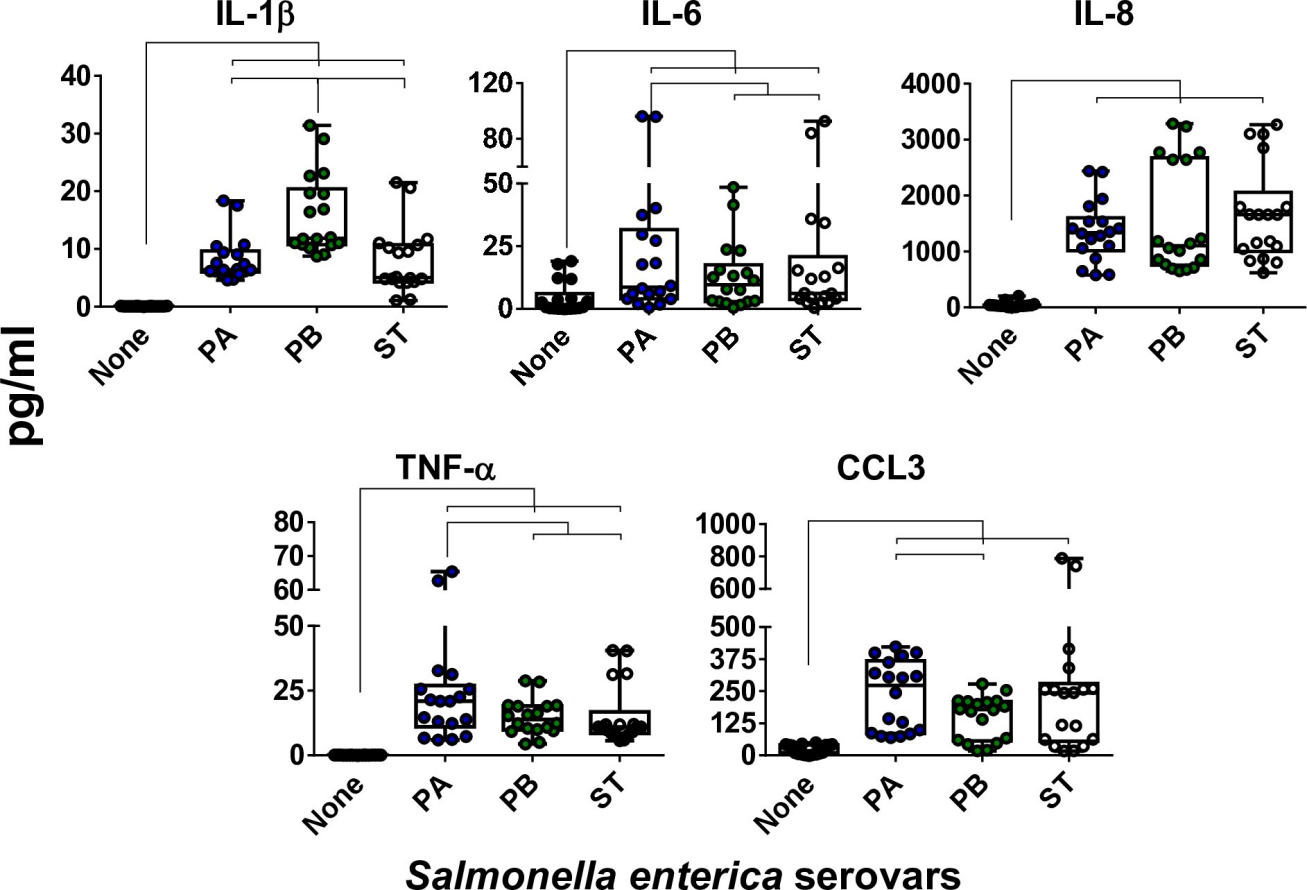
Cytokine production by the 3-D model cells after stimulation with three different *Salmonella* enterica serovars. 3-D model cells were left untreated (none) or exposed to either *Salmonella enterica* serovar Paratyphi A (PA), Paratyphi B (PB), or Typhi (ST) strains. After 4 hours, the levels of IL-1β, IL-6, IL-8, TNF-α and CCL3 cytokines in the culture supernatants were measured by Meso Scale Discovery (MSD) multiplex-assays. Bar graphs extend from the 25^th^ to 75^th^ percentiles; the line in the middle represents the median of the pooled data. The whiskers delineate the smallest to the largest value. The data represent up to 8 individual experiments for each of the *Salmonella* strains with 2 or 3 replicates in each experiment. Horizontal lines represent significant differences (*P*<0.05) between the indicated culture conditions.

**Fig 2:**
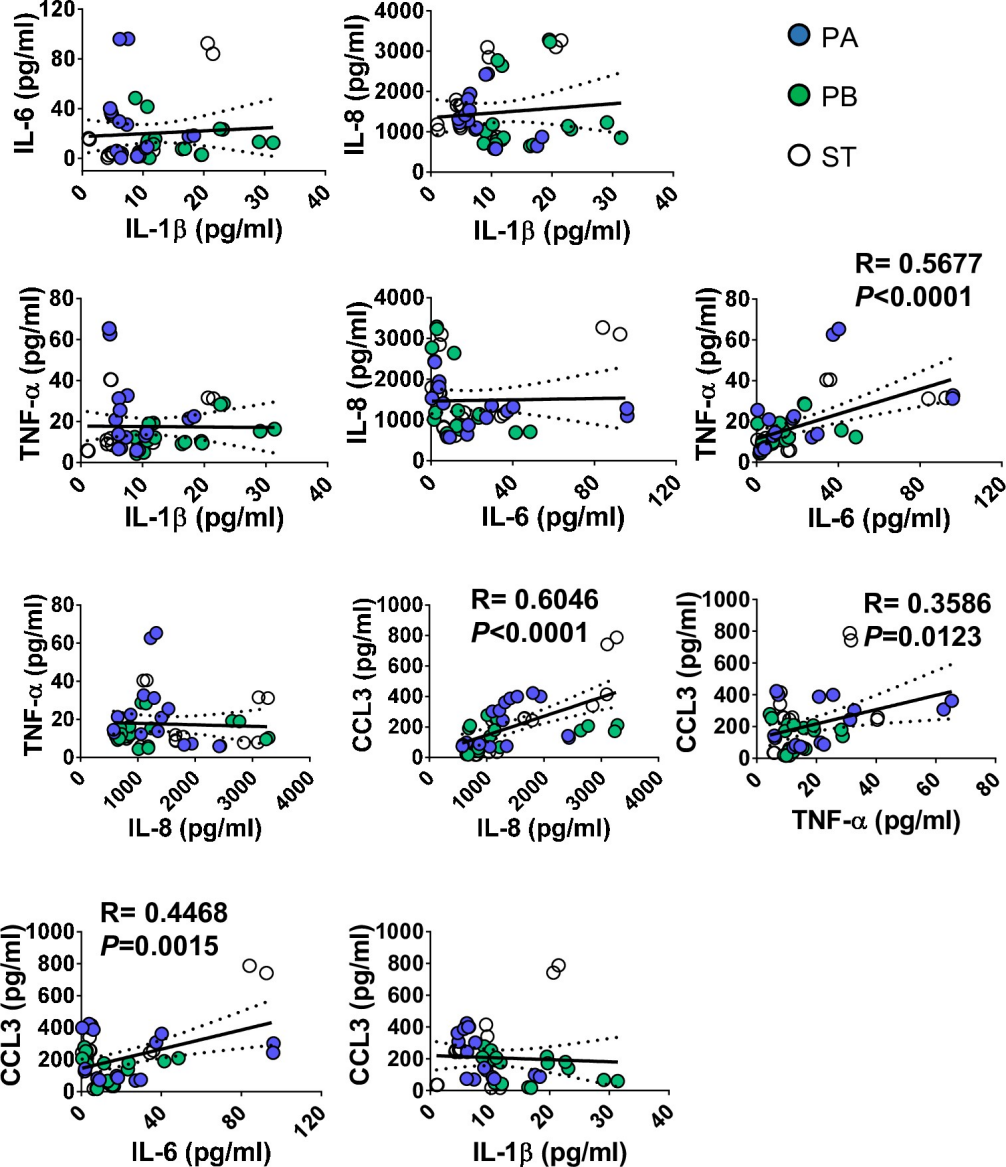
Correlation among cytokine/chemokine levels elicited by exposure to three different *Salmonella* enterica serovars. 3-D organotypic model cells were left untreated (none) or exposed to either *Salmonella enterica* serovar Paratyphi A (PA), Paratyphi B (PB), or Typhi (ST) strains. After 4 hours, the levels of IL-1β, IL-6, IL-8, TNF-α and CCL3 cytokines in culture supernatants were measured by Meso Scale Discovery (MSD) multiplex-assays. Correlations used the Pearson Product Moment tests. The data represent up to 8 individual experiments for each of *Salmonella* strains with 2 or 3 replicates in each experiment. Solid lines represent the trendlines. The correlation coefficient “R” and “*P*” values are shown when the correlations were significant. *P* values of <0.05 were considered statistically significant. Dashed lines represent 95% confidence intervals.

To investigate how the host recognizes distinct *Salmonella* serovars that share a high degree of homology, we took advantage of a multicellular three-dimensional (3-D) organotypic model of the human intestinal mucosa, which was developed and characterized by our group [34–39], and three genetically similar serovars, namely PA, PB, and ST. The 3-D model was comprised of primary human lymphocytes, fibroblasts, endothelial cells, and a human epithelial cell line [34–39]. These epithelial cells consist of a human adenocarcinoma enterocyte cell line (*i*.*e*., HCT-8) that was initially derived from the junction of the small and large bowel [40]. This 3-D model has unique characteristics with a close structural and functional resemblance to the human intestinal mucosa. In this organotypic 3-D model, the epithelial cell line behaves as a multipotent progenitor cell that gives rise to functional and highly differentiated cells from multiple lineages (*i*.*e*., absorptive enterocyte, goblet, and M cells)[34–36, 38]. Our focus was on the early interactions between bacteria and cells from the 3-D organotypic model. Cells were exposed (or not) to *Salmonella* serovars PA, PB, and ST. After 4 hours of incubation, the supernatants were collected and used to measure cytokines (i.e., IL-1β, IL-6, IL-8, CCL3, and TNF-α). We found that exposure to any of the *Salmonella* strains, albeit at different levels, resulted in increases in cytokine secretion compared to negative controls (**Fig 1**). Interestingly, we observed different patterns of secretion in the cytokines elicited by each *Salmonella* strain. Exposure to PB induced a significantly higher secretion of IL-1β compared to that of cultures exposed to PA and ST (**Fig 1**). The exposure to PA prompted a significantly higher secretion of IL-6 and TNF-α compared to cultures exposed to PB and ST (**Fig 1**). The exposure to PA also triggered a significantly higher secretion of CCL3 compared to 3-D organotypic models exposed to PB, and a similar trend for ST was observed (**Fig 1**). In contrast, no significant differences in the secretion of IL-8 were observed among the strains (**Fig 1**). Moreover, we found a significant correlation between the secretion of IL-8, a cytokine predominantly secreted by intestinal epithelial cells (IECs), and CCL3, which are chemokines/cytokines predominantly produced by immune cells (**Fig 2**). We also observed a significant correlation between the secretion of CCL3, and either IL-6 or TNF-α, which are pro-inflammatory cytokines. Thus, the inflammatory effects of PA, PB and ST serovars might be mediated, at least in part, through the differential regulation of cytokines/chemokines secreted by epithelial and immune cells.

### Impact of immune cells on the production of cytokines

**Fig 3:**
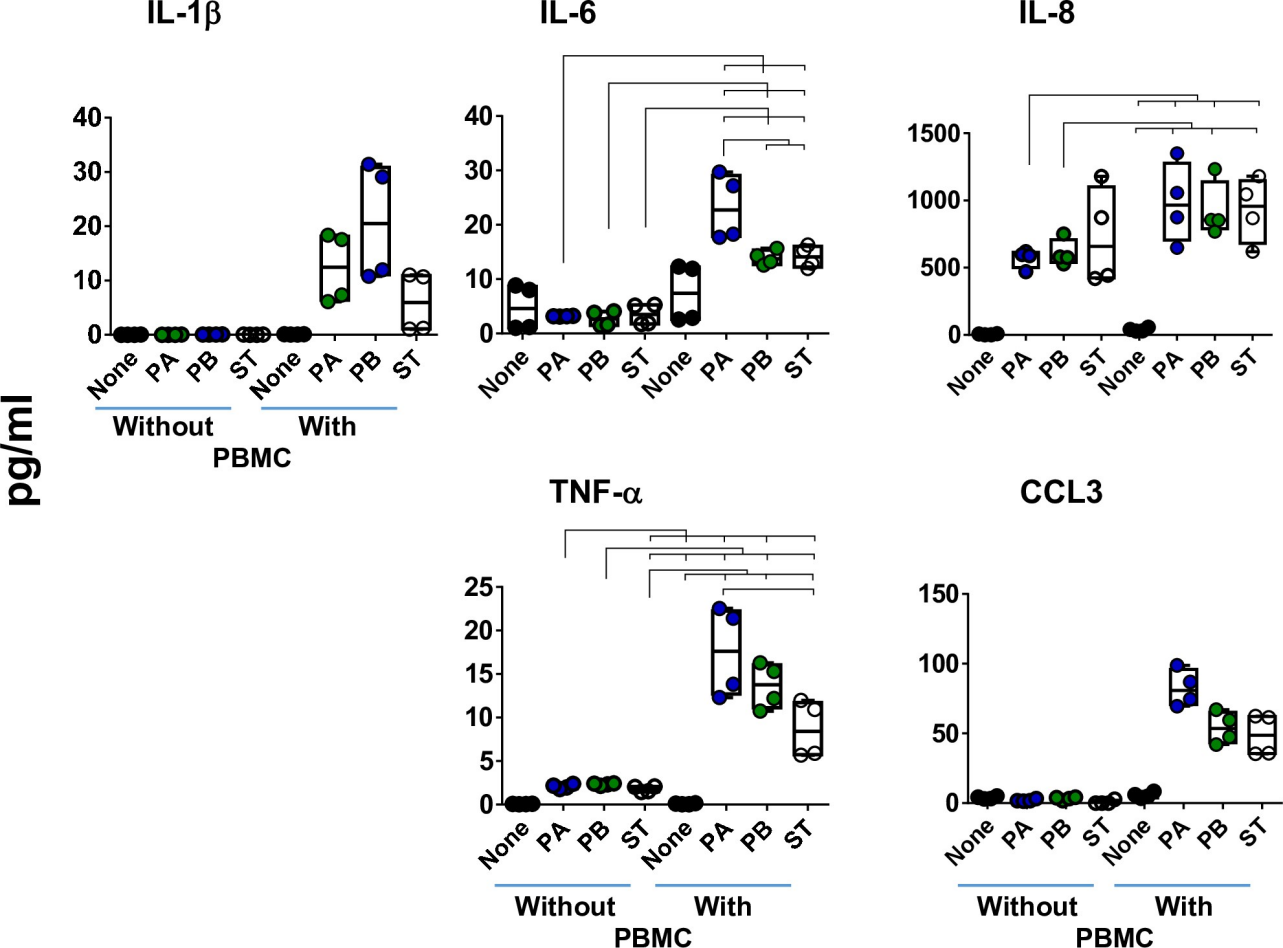
Impact of the presence of immune cells on cytokine production following exposure to three different *Salmonella* enterica serovars. Immune cells (i.e., PBMC) were added, or not, to the culture at 6 ± 1 and 13 ± 1 days after the setting up of the 3-D organotypic model. Once the 3-D organotypic models reached maturity (~17 days after initiation of the culture), they were exposed or not to either *Salmonella enterica* serovar Paratyphi A (PA), Paratyphi B (PB), or Typhi (ST) strains. After 4 hours, the levels of IL-1β, IL-6, IL-8, TNF-α and CCL3 cytokines in the culture supernatants were measured by Meso Scale Discovery (MSD) multiplex-assays. Bar graphs extend from the 25^th^ to 75^th^ percentiles; the line in the middle represents the median of the pooled data. The whiskers delineate the smallest to the largest value. The data represent up to 2 individual experiments for each of *Salmonella* strains with 2 replicates each. Horizontal lines represent significant differences (*P*<0.05) between the indicated culture conditions. Complete list of *P* values is shown in **S1 Table**.

It is well known that Mϕ play an essential role in the host response to *Salmonella* infection [3, 41], and that the chemokine CCL3 is critical in the regulation and recruitment of leukocytes (i.e., Mϕ and PMN) [42–44]. Moreover, Mϕ release and promote the secretion of pro-inflammatory cytokines, such as IL-1β, IL-6, and TNF-α, by other immune cells [45, 46]. Thus, to evaluate the role of Mϕ and other immune cells in the 3-D organotypic model, we next performed “gain and loss” studies to define the importance of immune cells (*i*.*e*., lymphocytes and Mϕ) (peripheral blood mononuclear cells, PBMC) in the production of cytokines. To this end, 3-D organotypic models were built without or with lymphocytes/monocytes-enriched PBMC. After ~17 days, the organotypic models were exposed, or not, to PA, PB or ST strains. After 4 hours of stimulation, the supernatants were collected and used to measure the secretion of IL-1β, IL-6, IL-8, CCL3, and TNF-α cytokines/chemokines. Regardless of the bacterial strain, the secretion of IL-1β and CCL3 was only observed in the supernatants from 3-D models containing immune cells (*i*.*e*., PBMC) (**Fig 3**). We also observed that the secretion of IL-6 and TNF-α was significantly higher in supernatants from organotypic models containing PBMC than in supernatants from 3-D organotypic models built without PBMCs (**Fig 3**). Finally, exposure to PA and PB induced a significantly higher secretion of IL-8 in the supernatants from 3-D organotypic models containing immune cells as compared to supernatants from 3-D models built without PBMC (**Fig 3**). No significant difference was found for ST (**Fig 3**) (*P* = 0.1762, ST with vs. ST without PBMC). Taken together, these results suggest that immune cells located at intestinal mucosa contribute substantially to the production of multiple chemokines/cytokines in responses to PA, PB, and ST infections.

### Activation of Mϕ by *Salmonella* strains PA, PB, and ST

**Fig 4:**
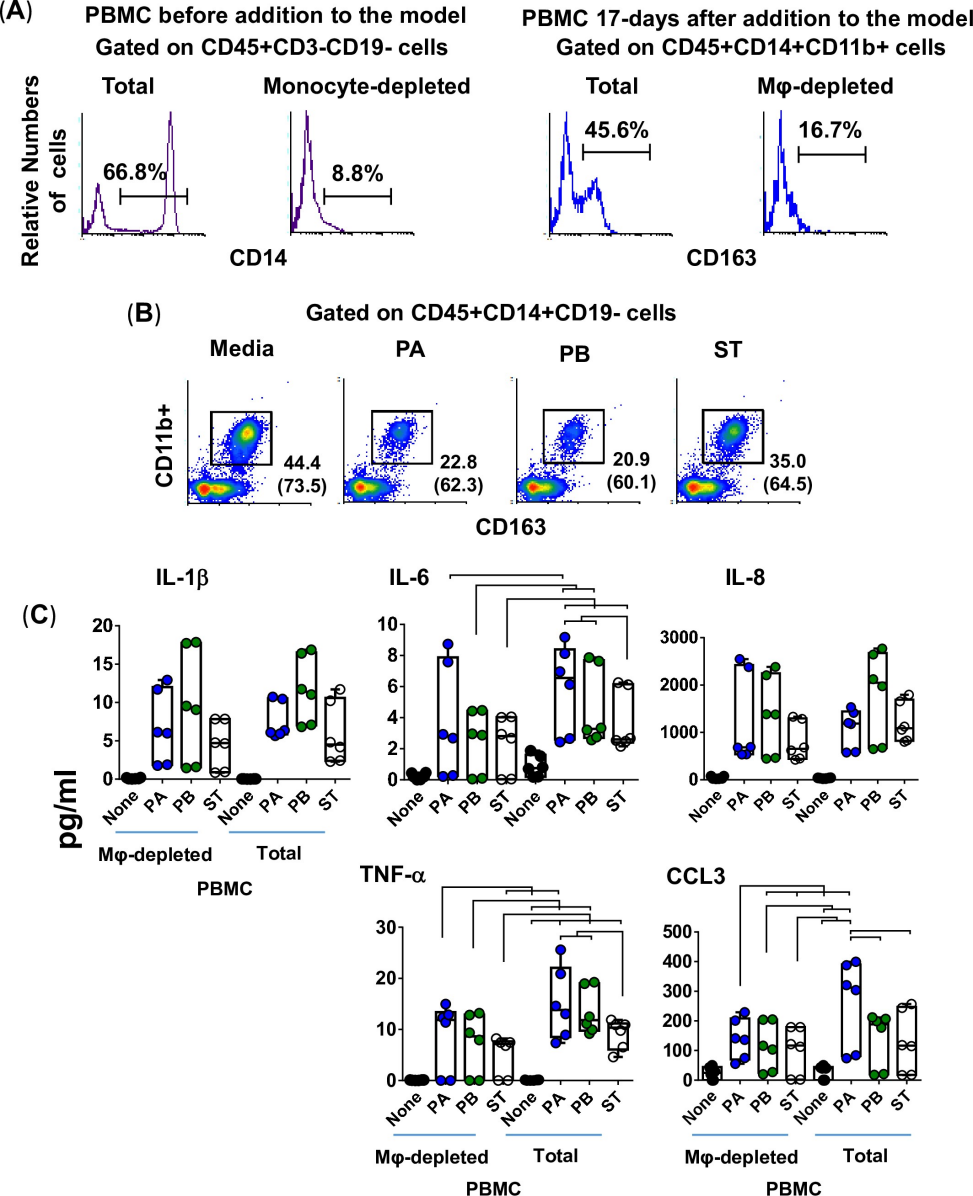
Impact of macrophages on the production of cytokines following exposure to three different *Salmonella* enterica serovars. Macrophages were removed from the PBMC by using adherence to gelatin technique, and the macrophage-depleted PBMC (mainly lymphocytes) were added to the culture at 6 ± 1 and 13 ± 1 days after the setting up of the 3-D organotypic model. Whole PBMC (lymphocytes/monocytes) was used as a control. (**A**) Flow cytometry analysis. **Left Panels**, Total and monocyte-depleted PBMC before addition to the model. Cells were gated on monocytes (CD45+CD3-CD19-CD14+). **Right panels**, Total and macrophage-depleted PBMC (Mφ-depleted) 17-days after addition to the model. Macrophages were gated based on their light scatter characteristics and specific lineage differentiation markers: CD45+CD14+CD11b+CD163+. (**B**) 3-D organotypic model cells were left untreated (none) or exposed to either *Salmonella enterica* serovar Paratyphi A (PA), Paratyphi B (PB), or Typhi (ST) strains. After 4 hours, constructs and supernatants were collected. Constructs were used to isolate cells and measure the levels of resident macrophages in various culture conditions by flow cytometry. Numbers correspond to the % positive cells, followed by mean fluorescence intensity (MFI) in parenthesis (*x*-axis). (**C**) Supernatants were used to measure the levels of IL-1β, IL-6, IL-8, TNF-α and CCL3 cytokines by Meso Scale Discovery (MSD) multiplex-assays. Bar graphs extend from the 25^th^ to 75^th^ percentiles; the line in the middle represents the median of the pooled data. The whiskers delineate the smallest to the largest value. The data represent 3 individual experiments for each of *Salmonella* strains with 2 replicates per experiment. Horizontal lines represent significant differences (*P*<0.05) between the indicated culture conditions. Complete list of *P* values is shown in **S2 Table**.

To characterize the role of Mϕ in the secretion of these cytokines, we depleted Mϕ from PBMC by using the adherence to agarose technique [47, 48], and the Mφ-depleted PBMC were added during the construction of the 3-D organotypic models. Organotypic models built with whole PBMC (Total PBMC) were used as controls. Using this technique, monocyte depletion was >85% by flow cytometry analysis. A representative experiment is shown in **Fig 4A**, left 2 panels. After 17 days in culture, constructs from the 3-D models were collected, disaggregated by collagenase, and single cell suspensions used for surface marker staining (CD11b, CD14, CD19, CD45, and CD163). Flow cytometry analysis showed a reduction of 50–60% in Mϕ present in the models built with Mϕ-depleted PBMC compared to that of the models built with whole PBMC. A representative experiment is shown in **Fig 4A**, right 2 panels.

Alternatively, or concomitantly, after 17 days in culture, the cells from the 3-D organotypic models were left untreated (none) or were exposed to either PA, PB or ST. After 4 hours, the constructs and supernatants were collected. Single cells were isolated from the constructs as described above. Flow cytometric analyses showed lower numbers of Mϕ (*i*.*e*., CD45+CD14+CD19-CD11b+CD163+ cells) in the constructs exposed to *Salmonella* strains compared with the control constructs with media only (**Fig 4B**). Constructs exposed to PA and PB had higher decreases in Mϕ than did the constructs exposed to ST. We hypothesized that more Mϕ died in 3-D organotypic models exposed to PB and PA as compared to 3-D organotypic models exposed to ST. We then used supernatants from constructs exposed to the *Salmonella* serovars to measure the levels of IL-1β, IL-6, IL-8, TNF-α, and CCL3. We observed that regardless of the *Salmonella* strain, the secretion of IL-6 and TNF-α was higher in supernatants from organotypic models containing whole PBMC than in those from 3-D organotypic models built with Mϕ-depleted PBMC (**Fig 4C**). No changes in the secretion of IL-1β were observed among the strains when comparing supernatants from the 3-D models built with whole or Mϕ-depleted PBMC (**Fig 4C**). Interestingly, the secretion of CCL3 was higher in supernatants from models exposed to PA, but not to PB and ST, when comparing cultures with whole PBMC to cultures with Mϕ-depleted PBMC (**Fig 4C**). Thus, Mϕ were required in order to observe a predominant secretion of IL-6 and TNF-α in response to PA, PB and ST infection, and CCL3 in response to PA.

**Fig 5:**
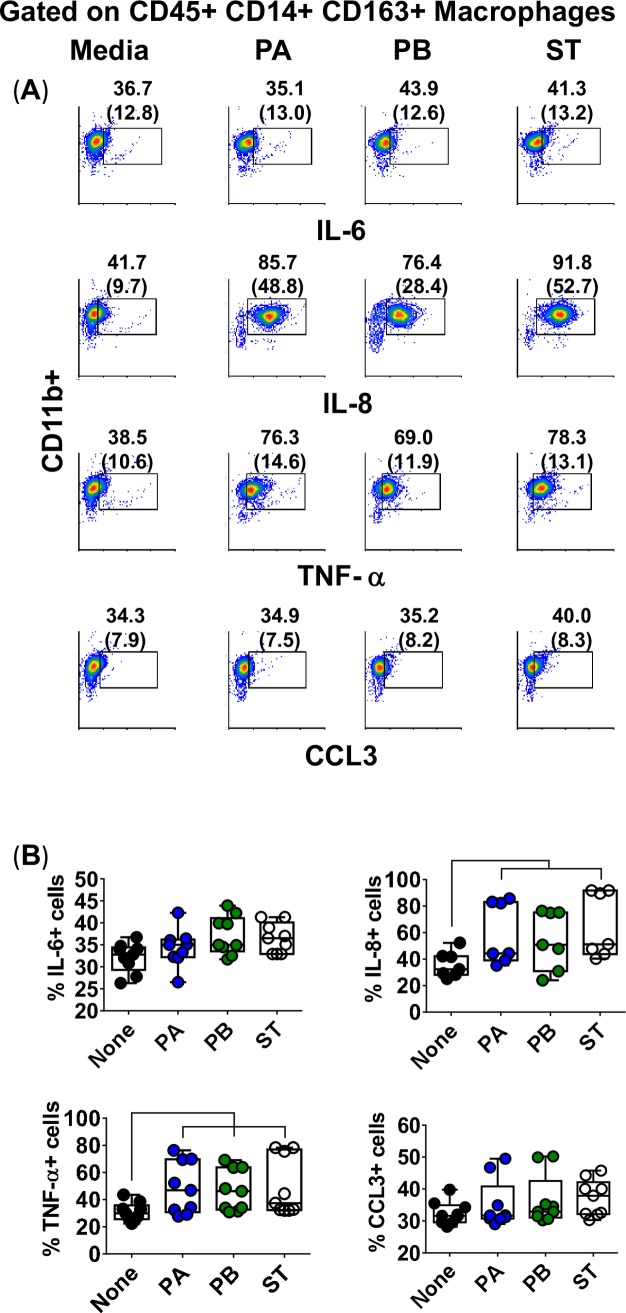
Macrophage expression of intracellular cytokines after stimulation with different *Salmonella* strains. 3-D organotypic models were exposed, or not, to either *Salmonella enterica* serovar Paratyphi A (PA), Paratyphi B (PB), or Typhi (ST) strains. After 4 hours, tissues were collected, disaggregated, and used for surface and intracellular staining. (**A**) Levels of IL-6, IL-8, TNF-α and CCL3 intracellular cytokines on macrophages were analyzed by flow cytometry. Macrophages were gated based on their scatter characteristics and specific lineage differentiation markers: CD45+ CD14+ CD163+ CD11b+. Numbers correspond to the % positive cells with mean fluorescence intensity (MFI) in parenthesis (*x*-axis). (**B**) Bar graphs extend from the 25^th^ to 75^th^ percentiles; the line in the middle represents the median of the pooled data. The whiskers delineate the smallest to the largest value. The data represent 3 individual experiments with up to 4 replicates in each experiment. Horizontal lines represent significant differences (*P*<0.05) between the indicated culture conditions.

**Fig 6:**
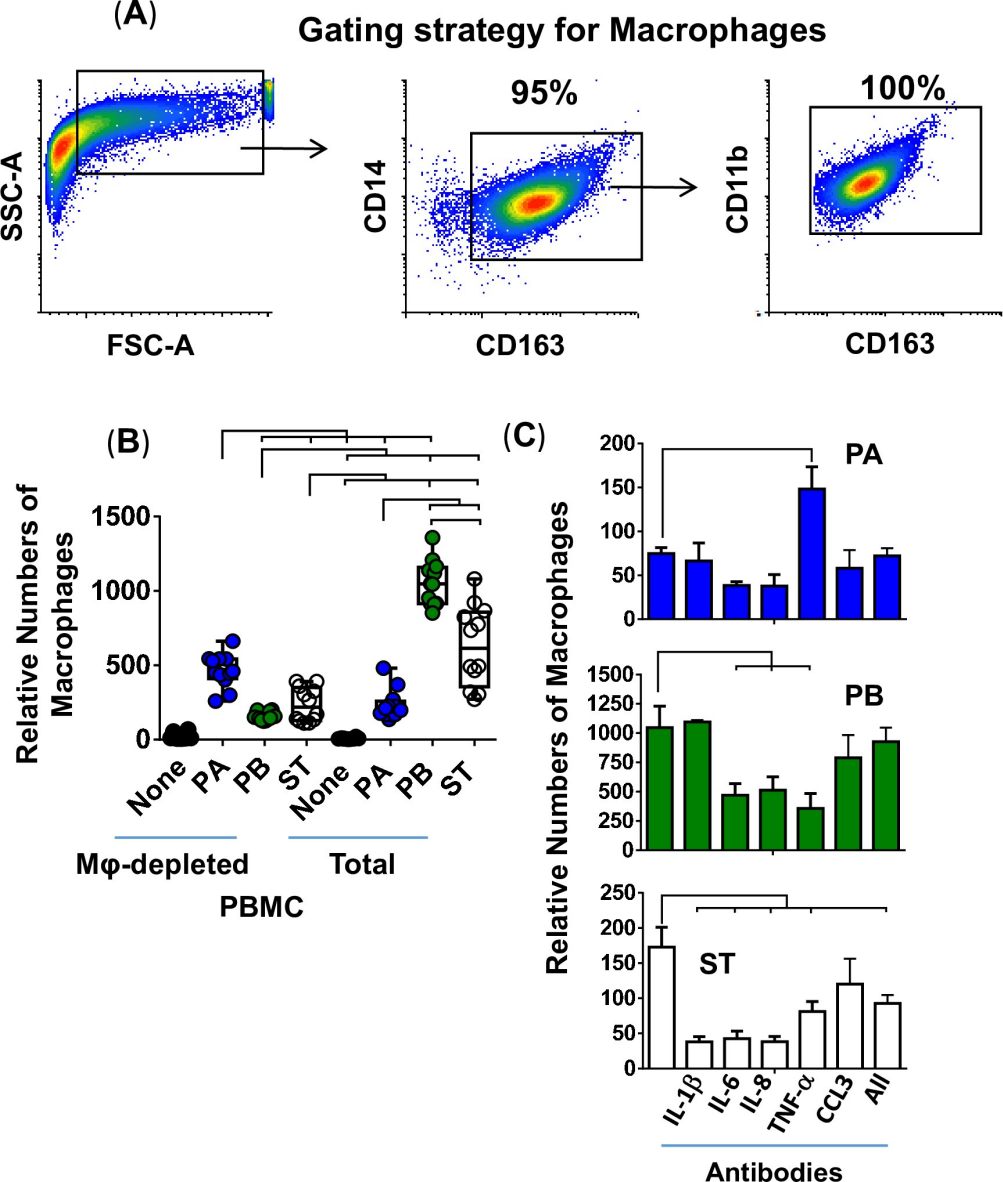
Role of cytokines/chemokines elicited following exposure to different *Salmonella* strains on macrophage migration. Macrophages were generated from the human monocyte cell line U937 using a phorbol 12-myristate 13-acetate protocol. (**A**) Gating strategy for macrophage analysis by flow cytometry. Macrophages were gated based on their light scatter characteristics and specific lineage differentiation markers: CD45+ CD14+ CD163+ CD11b+. Numbers correspond to the % positive cells. (**B**) 3-D organotypic models built with whole (Total) or macrophage-depleted (Mφ-depleted) PBMC were exposed or not to either *Salmonella enterica* serovar Paratyphi A (PA), Paratyphi B (PB), or Typhi (ST) strains. After 4 hours, supernatants were collected and used to stimulate macrophage migration in a trans-well system. Bar graphs extend from the 25^th^ to 75^th^ percentiles; the line in the middle represents the median of the pooled data. The whiskers delineate the smallest to the largest value. The data represent 4 individual experiments for each of *Salmonella* strains, each experiment with 3 replicates. Complete list of *P* values is shown in **S4 Table**. (**C**) Neutralizing monoclonal antibodies against IL-6, TNF-α, CCL3 or combined (All) were used for blocking experiments. Macrophages exposed to supernatants from a culture containing bacteria without or with antibodies are shown. Values represent means ± SE of one independent experiment with 3 replicates. Horizontal lines represent significant differences (*P*<0.05) between the indicated culture conditions.

To confirm that Mϕ were the source of the cytokines, we next measured the intracellular expression of IL-6, IL-8, TNF-α, and CCL3 by flow cytometry in cells isolated from 3D organotypic models to which PBMC were added. Although Mϕ constitutively expressed baseline levels of these cytokines (i.e., media only), expression of IL-8 and TNF-α were higher both per cell (mean fluorescence intensity), and in frequency (%) in the cultures exposed to the *Salmonella* strains than to controls (media) (**Fig 5**). IL-6 and CCL3 expression, though present, did not or only marginally increased after exposure to *Salmonella* strains as compared to the control (**Fig 5**). These results were further confirmed via Mϕ derived from the human monocyte cell line U937 obtained using phorbol 12-myristate 13-acetate [49]. This process resulted in >90% of cells expressing CD14+CD163+CD11b+ markers, a phenotype characteristic of resident Mϕ and consistent with one expressed by the Mϕ in the organotypic model [50, 51] (**Fig 6A**). For these experiments, U937-differentiated Mϕ were cultured with supernatants from 3-D organotypic models built with whole (Total) PBMC and exposed or not to either PA, PB, or ST strains. After 4 hours, cells were collected and used to measure viability by Trypan blue exclusion test, or to perform flow cytometric assays to analyze intracellular cytokine expression. Like for Mϕ isolated from the organotypic models, only IL-8 and TNF-α expression were markedly increased after exposure to *Salmonella* strains as compared to controls (media) (**S1B–S1E Fig**).

### Macrophage migration after exposure to PA, PB, and ST

To further explore the Mϕ functionality, we next performed experiments to evaluate the effects of the presence of Mϕ on cytokine/chemokine secretion and its impact on their migration. To this end, organotypic models built with whole or Mϕ-depleted PBMC were exposed or not to either PA, PB or ST strains. After 4 hours, the supernatants were collected and used to stimulate Mϕ migration over a trans-well system. Mϕ were differentiated as described above from the U937 cell line (**Fig 6A**). We found that the migration of Mϕ was higher in the presence of supernatants from models exposed to PB and ST than their migration in the presence of supernatants from models exposed to PA when the models were built with whole PBMC (**Fig 6B & S2 Fig**). Interestingly, the migrations observed when Mϕ were exposed to supernatants from models built with Mϕ-depleted PBMC and exposed to PA were increased, while the migrations when exposed to PB and ST supernatants were significantly decreased (**Fig 6B**). Thus, although one cannot assert that Mϕ present in the organotypic model are the only cells secreting chemokines, these results suggest that resident Mϕ might play a role in regulating the patterns of migration of circulating Mϕ into the local microenvironment. While soluble factors produced in response to PB and ST infections in cultures with Mϕ led to upregulation of Mϕ migration, soluble factors in response to PA infection mediated downregulation of Mϕ migration as compared to cultures with Mϕ-depleted PBMC.

To determine whether IL-1β, IL-6, IL-8, TNF-α, or CCL3 were involved in the signaling of Mϕ, we depleted these cytokines/chemokines using monoclonal antibodies. Depletion of IL-1β, IL-6, IL-8, and TNF-α, but not CCL3, decreased the migration of Mϕ exposed to supernatants from ST-cultures (**Fig 6C**). Anti-IL-6, -IL-8 or -TNF-α antibodies also decreased the Mϕ chemotactic effect of supernatants obtained from the PB-exposed cultures (**Fig 6C**). Remarkably, blocking of TNF-α induced an increase in the Mϕ chemotactic activity of supernatants derived from the PA-exposed cultures (**Fig 6C**). Finally, blocking of CCL3 had only modest effects on Mϕ migration after exposure to supernatants obtained from ST- and PB-exposed cultures. These results suggest that the signaling of Mϕ migration is dependent on infection with particular *Salmonella* serovars.

**Fig 7:**
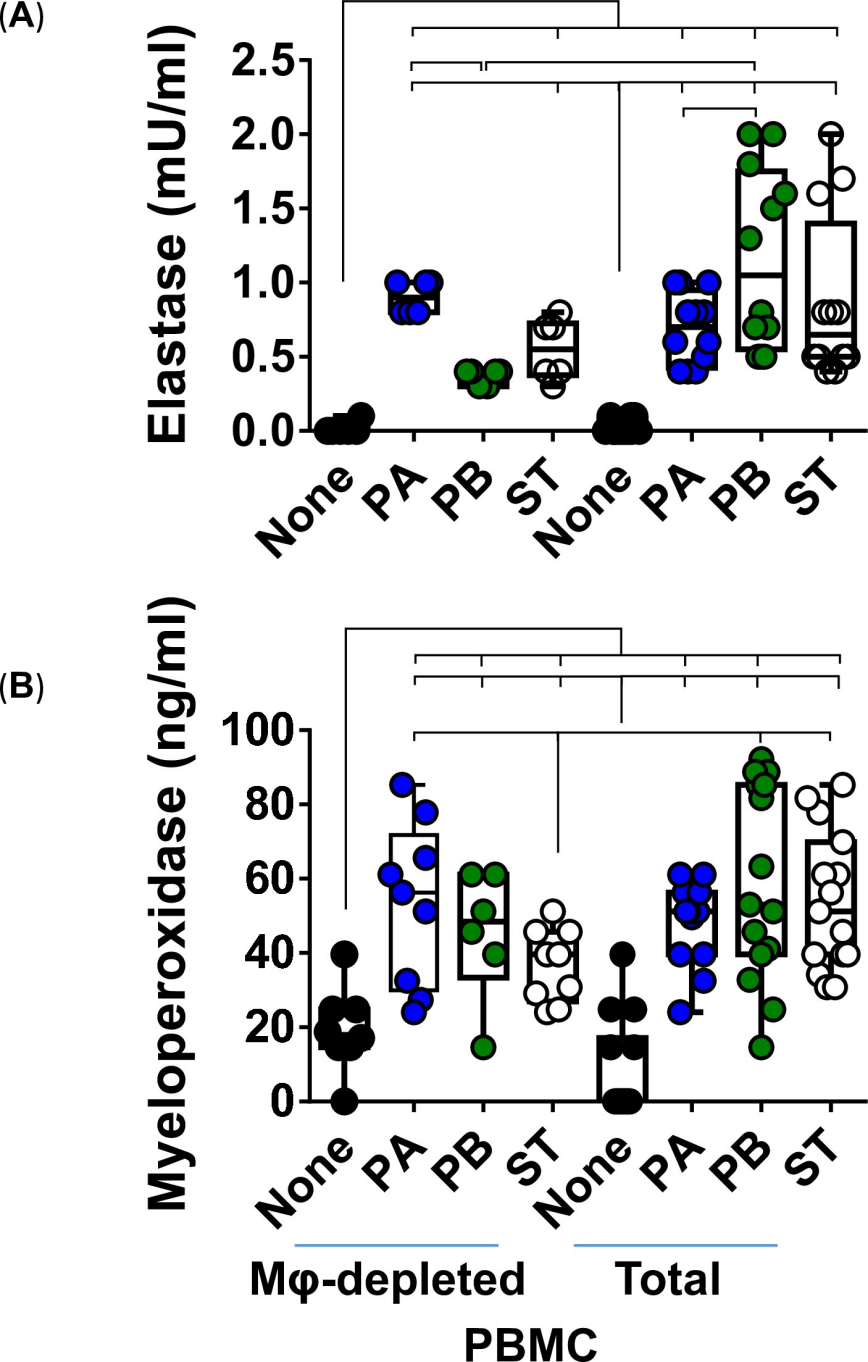
Production of antibacterial products by macrophages after stimulation with different *Salmonella* Typhi strains. 3-D organoids built with whole (Total) or macrophage-depleted (Mφ-depleted) PBMC were exposed, or not, to either *Salmonella enterica* serovar Paratyphi A (PA), Paratyphi B (PB), or Typhi (ST) strains. After 4 hours, supernatants were collected and used to measure (**A**) elastase and (**B**) myeloperoxidase by ELISA. Bar graphs extend from the 25^th^ to 75^th^ percentiles; the line in the middle represents the median of the pooled data. The whiskers delineate the smallest to the largest value. The data represent up to 5 individual experiments for each of *Salmonella* strains with 3 replicates each. Horizontal lines represent significant differences (*P*<0.05) between the indicated culture conditions. Complete list of *P* values is shown in **S5 Table**.

To further investigate the functionality of Mϕ, we examined their bactericidal ability by measuring the release of elastase and myeloperoxidase (MPO) after exposure to PA, PB, and ST strains. The production of elastase and MPO are part of the antimicrobial arsenal of Mϕ to fight infection. After 4 hours of infection, supernatants from the cultures with whole and Mϕ-depleted PBMC were collected and used to measure the release of elastase and MPO by ELISA. The production of elastase and MPO was similar between PA-derived supernatants from the organotypic models built with whole (total) and Mϕ-depleted PBMC (**Fig 7**). It is worth noting that the elastase levels were significantly higher or showed a significant trend in supernatants from the PB (*P* = <0.0001) and ST (*P* = 0.0785) cultures, when the models were built with whole as compared to the organotypic models built with Mϕ-depleted PBMC (**Fig 7**). Thus, Mϕ are more prone to produce elastase after exposure to PB and ST than after exposure to PA.

**Fig 8:**
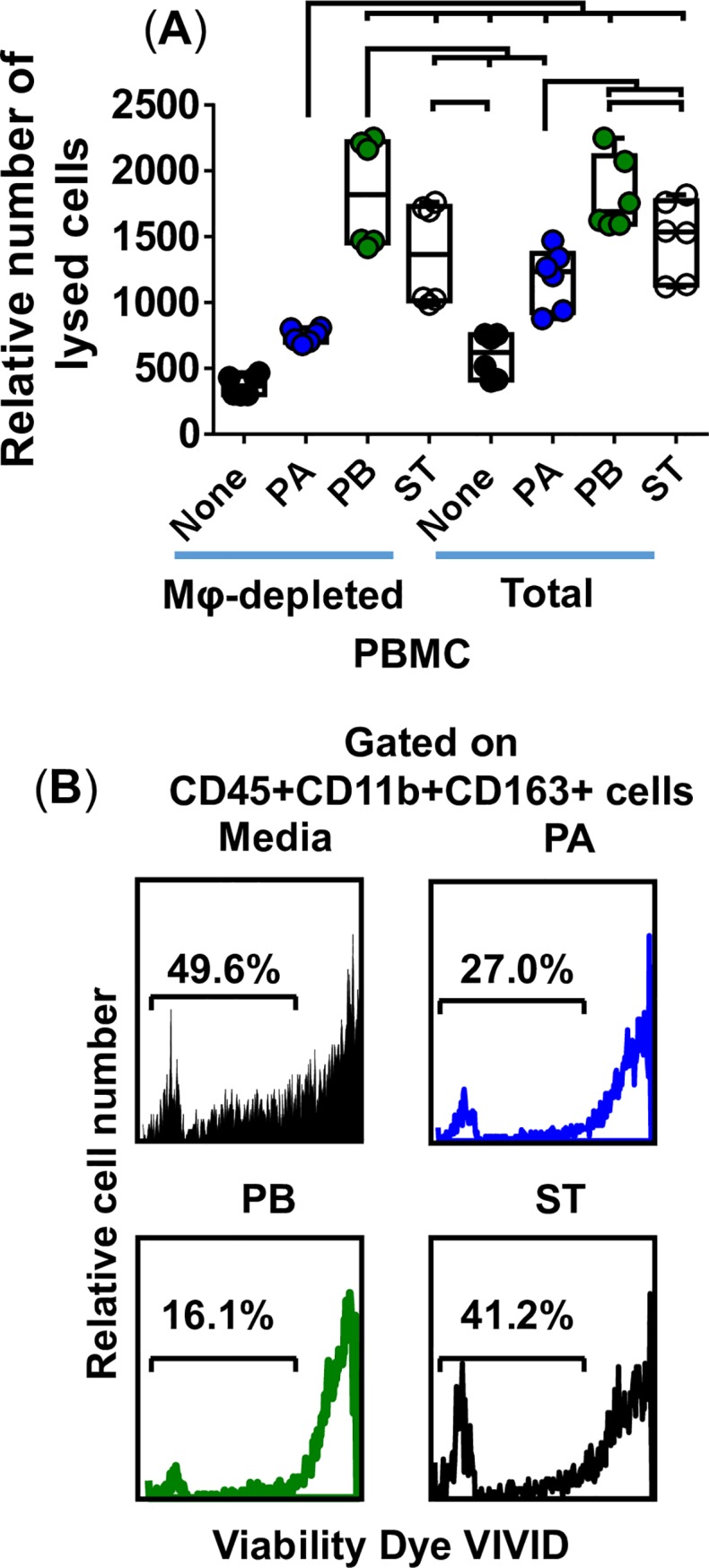
Effect of immune cells on cell viability. 3-D organotypic models built with the whole (Total) or macrophage-depleted (Mφ-depleted) PBMC were exposed or not to either *Salmonella enterica* serovar Paratyphi A (PA), Paratyphi B (PB), or Typhi (ST) strains. (**A**) After 4 hours, supernatants were collected and used to measure cell viability by using a commercial lactate dehydrogenase (LDH) assay. Bar graphs extend from the 25^th^ to 75^th^ percentiles; the line in the middle represents the median of the pooled data. The whiskers delineate the smallest to the largest value. The data represent 2 individual experiments for each of *S*. Typhi strains, each experiment with 3 replicates. Horizontal lines represent significant differences (*P*<0.05) between the indicated culture conditions. Complete list of P values is shown in **S6 Table**. (**B**) Macrophage viability detected using violet fluorescent dye ViViD. After 4 hours, tissues from 3-D models built with whole PBMC were collected, disaggregated, and used to perform flow cytometry. Numbers represent the percentage (%) of positive cells in the live cell gate (ViViD negative).

We next evaluated cell viability in the presence or absence of Mϕ in the model. After 4 hours of infection, cell viability was evaluated by lactate dehydrogenase (LDH), a stable cytosolic enzyme that is released into cell supernatant upon cell lysis. Regardless of the presence of Mϕ, PB, and to lesser degree ST, infection induced the highest levels of cell killing. Interestingly, the levels of cell death in the PA cultures were higher in the organotypic models built with whole PBMC than in those built with Mϕ-depleted PBMC. Thus, while the levels of cell death in the PB and ST cultures encompassed multiple cell types (*e*.*g*., epithelial cells and immune cells), PA killing appears to be mainly restricted to Mϕ. Flow cytometry analysis of cell viability, based on dye VIVID, showed that Mϕ were more susceptible to death after exposure to PB than after exposure to PA or ST (**Fig 8B**). Therefore, although PA killing affected more Mϕ, these killings were lower in magnitude than those observed for PB but more noticeable than those for ST.

### Effect of Mϕ on PMN migration

**Fig 9:**
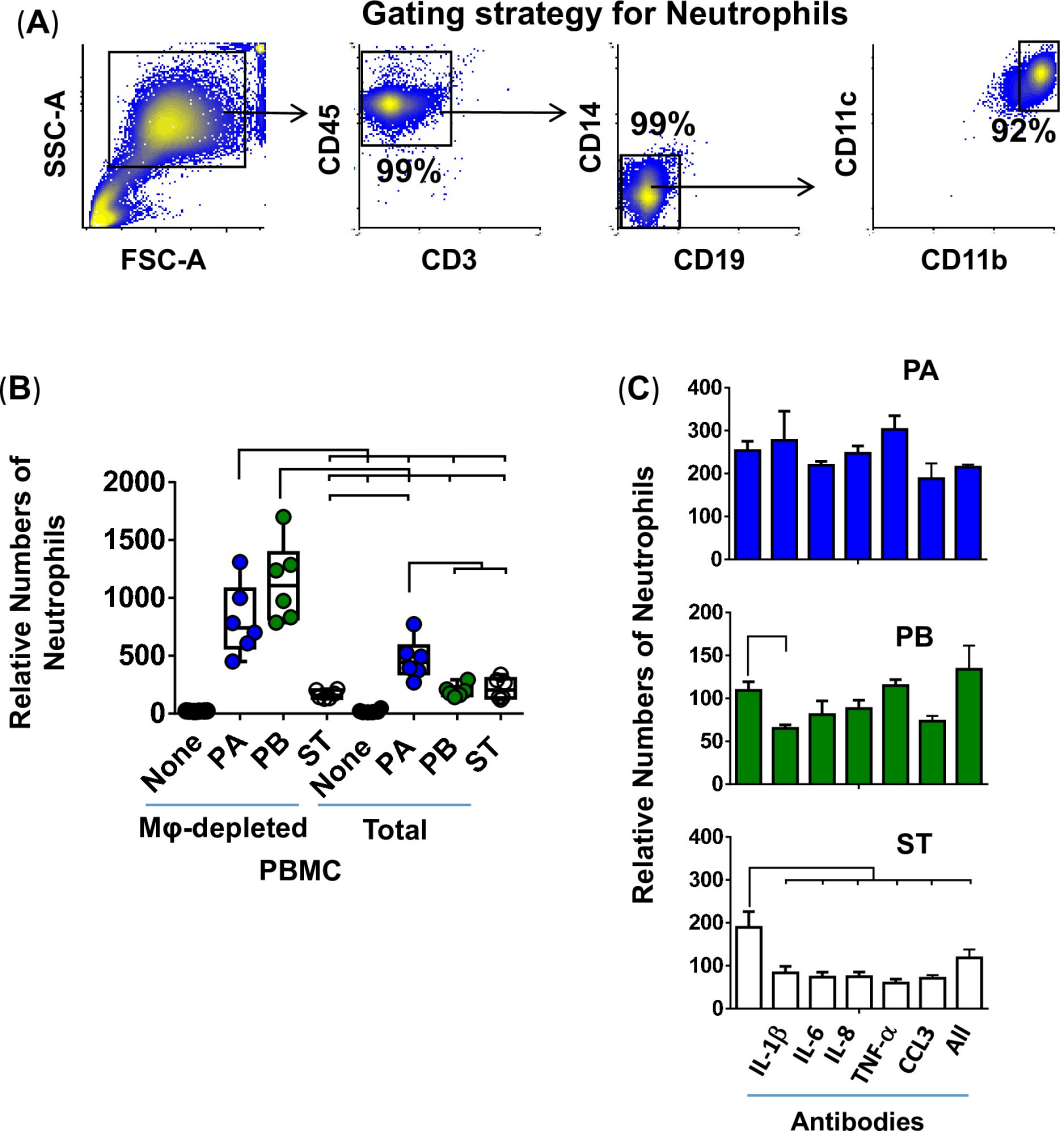
Effect of macrophages on PMN migration. PMN were isolated using a standard dextran-500 gradient technique. (**A**) Gating strategy for PMN analysis by flow cytometry. PMN were gated based on their light scatter characteristics and specific lineage differentiation markers (CD45+CD3-CD14-CD19-CD11b+CD11c+). Numbers correspond to the % positive cells. (**B**) 3-D organotypic models built with whole (Total) or macrophage-depleted (Mφ-depleted) PBMC were exposed or not to either *Salmonella enterica* serovar Paratyphi A (PA), Paratyphi B (PB), or Typhi (ST) strains. After 4 hours, supernatants were collected and used to stimulate PMN migration in a trans-well system. Bar graphs extend from the 25^th^ to 75^th^ percentiles; the line in the middle represents the median of the pooled data. The whiskers delineate the smallest to the largest value. The data represent 2 individual experiments for each of *Salmonella* strains with 3 replicates per experiment. Complete list of *P* values is shown in **S7 Table**. (**C**) Neutralizing monoclonal antibodies against IL-6, TNF-α or CCL3 or combined (All) were used for blocking experiments. PMN exposed to supernatants from a culture containing bacteria without or with antibodies are shown. Values represent means ± SE of one independent experiment with 3 replicates. Horizontal lines represent significant differences (P<0.05) between the indicated culture conditions.

Since during the early stages of *Salmonella* infection, PMN are recruited by chemokines released by resident cells, such as Mϕ [52], we next assessed whether, *in vitro*, the factors secreted by Mϕ were capable of modulating *Salmonella*-driven PMN migration. To this end, PMN were purified by a standard dextran-500 gradient technique [53] and were used to measure chemotaxis after exposure to cell-free supernatants from organotypic models exposed, or not, to PA, PB, or ST. The PMN purity was ~90% (**Fig 9A**). We found differing levels of PMN migration between infection conditions of organotypic models built with whole or Mϕ-depleted PBMC. Notably, exposure of purified PMN to supernatants from the PA and PB, but not ST, cultures built with whole PBMC exhibit significant reductions in PMN migration (**Fig 9B & S2 Fig**) compared to the cultures built with Mϕ-depleted PBMC (**Fig 9B**). Interestingly, no significant changes in PMN migration were observed between cultures with whole and Mϕ-depleted PBMC after exposure to supernatants from organotypic models containing ST (**Fig 9B**). These results strongly suggest the existence of feedback mechanisms between Mϕ and PMN during *Salmonella* infections.

Next, we performed blocking experiments to determine the impact of IL-1β, IL-6, IL-8, TNF-α, and CCL3 on PMN migration. To this end, organotypic models built with whole PBMC were exposed or not to PA, PB, or ST. After 4 hours, the supernatants were collected and used to stimulate PMN migration over a trans-well system. We found that depletion of IL-1β, IL-6, IL-8, TNF-α and CCL3 decreased the migration of PMN exposed to supernatants from ST-cultures (**Fig 9C**). Anti-IL-1β antibodies also decreased the PMN chemotactic effect of supernatants obtained from the PB-exposed cultures (**Fig 9C**). Besides, CCL3 showed a trend (*P* = 0.067) of blocking PMN chemotaxis triggered by supernatants containing PB, without reaching statistical significance, which could be attributable to the concentrations of both CCL3 and anti-CCL3 antibodies in the supernatants. Regardless of the neutralizing antibodies used, PMN migration was not blocked after exposure to supernatants obtained from the PA-exposed cultures (**Fig 9C**). These *in vitro* observations prompted us to speculate the existence of synergic and/or antagonistic effects between the expression of cytokine/chemokines such as IL-6, IL-8, TNF-α and CCL3 that determine PMN migration following exposure to various *Salmonella* serovars. It is also possible that other cytokines/chemokines not evaluated in these studies play differential roles in the PMN migration, which we could not observe since we did not use the appropriate blocking mAbs.

**Fig 10:**
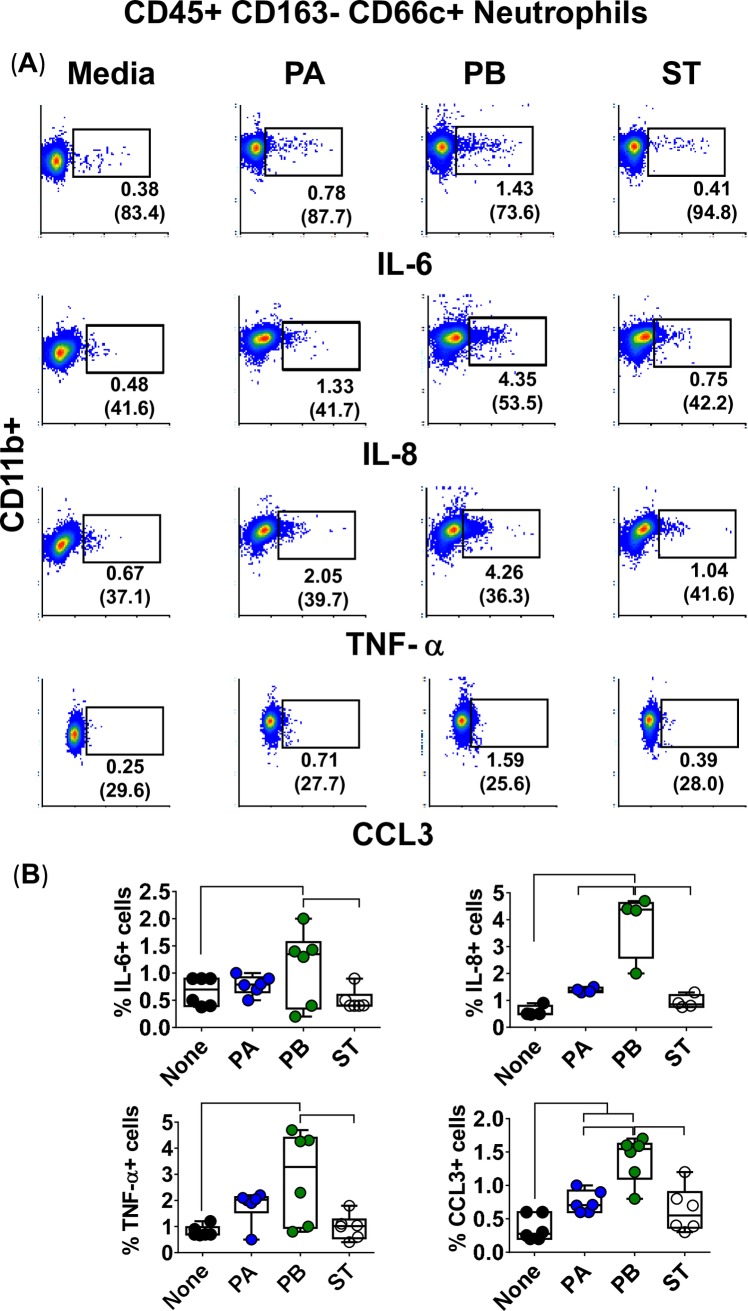
PMN expression of intracellular cytokines after stimulation with different *Salmonella* strains. 3-D organotypic models were exposed, or not, to either *Salmonella enterica* serovar Paratyphi A (PA), Paratyphi B (PB), or Typhi (ST) strains. After 4 hours, supernatants were collected and used to stimulate dextran-500-purified PMN. (**A**) After 4 hours of stimulation, PMN were surface and intracellular stained, and the levels of IL-6, IL-8, TNF-α and CCL3 intracellular cytokines measured by flow cytometry. PMN were gated based on their scatter characteristics and specific lineage differentiation markers: CD45+CD163-CD66c+. Numbers correspond to the % positive cells, followed by mean fluorescence intensity (MFI) in parenthesis (*x*-axis). (**B**) Bar graphs extend from the 25^th^ to 75^th^ percentiles; the line in the middle represents the median of the pooled data. The whiskers delineate the smallest to the largest value. The data represent 2 individual experiments with up to 3 replicates in each experiment. Horizontal lines represent significant differences (*P*<0.05) between the indicated culture conditions.

To study the association between PMN migration and their capacity to produce cytokines, we measured their intracellular expression of IL-6, IL-8, TNF-α, and CCL3 cytokines. Purified PMN were exposed to supernatants from PA, PB or ST cultures built with whole PBMC. After 4 hours, PMN were surface stained with CD3, CD14, CD19, CD45, CD163, and CD66c, and intracellularly with IL-6, IL-8, TNF-α and CCL3 mAbs and analyzed by flow cytometry. We found that PB induced higher levels of cytokines than either PA or ST (**Fig 10**). Although the frequency of PMN positive for IL-6, IL-8, TNF-α, and CCL3 is much lower than that for Mϕ, they might have a significant contribution to the overall production of IL-6, IL-8, TNF-α, and CCL3 during inflammation, since PMN are at higher frequency than Mϕ at sites of acute inflammation [54]. In conclusion, these results demonstrated that, as for Mϕ, PMN might be important producers of IL-6, IL-8, TNF-α, and CCL3 and their migration might require additional signals provided by bacteria or cytokines/chemokines produced by themselves or others to develop their functions.

### Activation of intestinal epithelial cells by *Salmonella* strains PA, PB and ST

**Fig 11:**
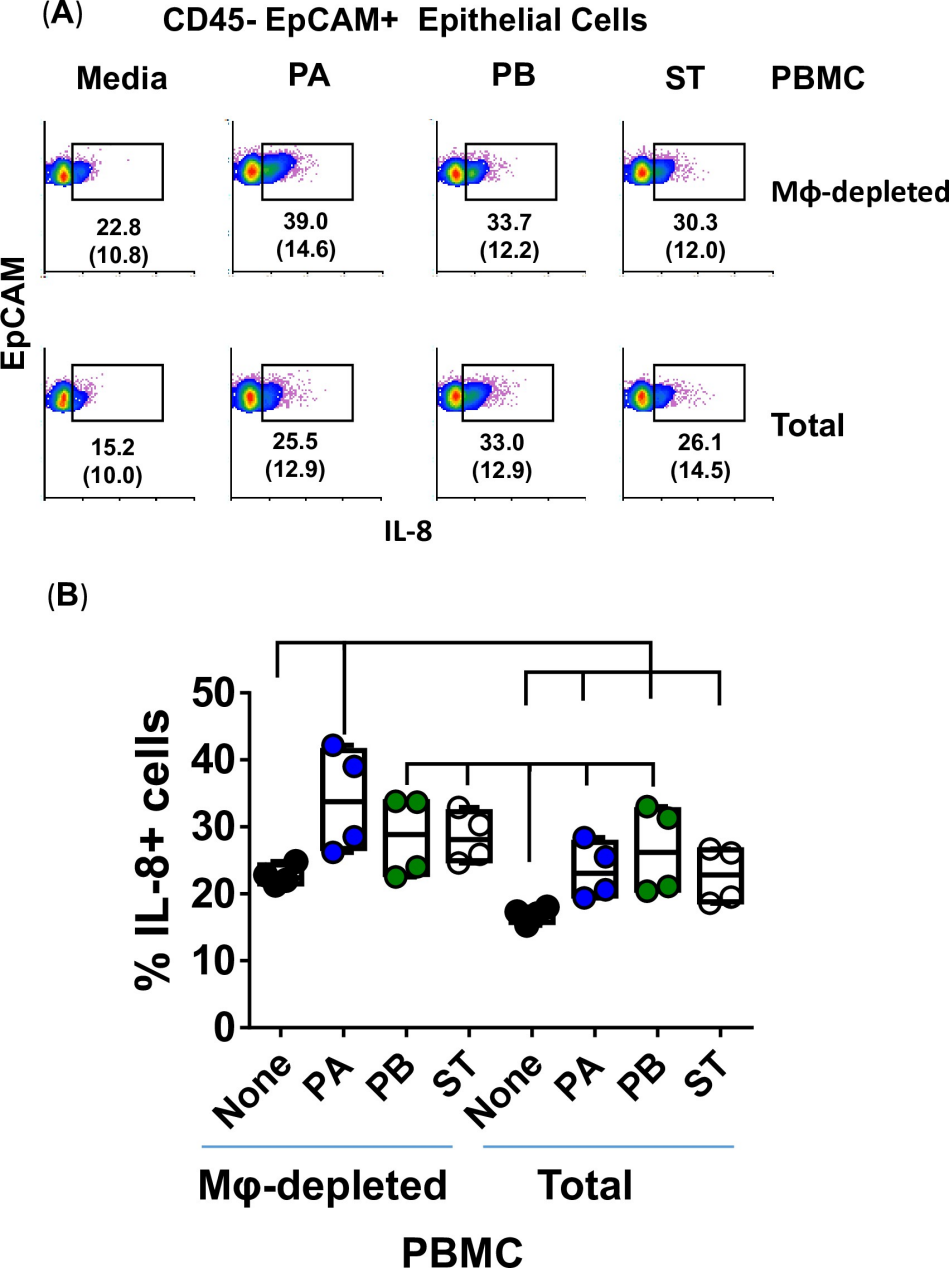
Epithelial cell expression of IL-8 after stimulation of the 3-D organotypic models with different *Salmonella* serovars. 3-D organotypic models built with whole (Total) or macrophage-depleted (Mφ-depleted) PBMC were exposed or not to either *Salmonella enterica* serovar Paratyphi A (PA), Paratyphi B (PB), or Typhi (ST) strains. (**A**) After 4 hours, tissues were collected, disaggregated, and used to perform IL-8 intracellular staining followed by flow cytometric analysis. Epithelial cells were gated based on their scatter characteristics and specific lineage differentiation markers: CD45-EpCAM+. Numbers correspond to the % positive cells, followed by mean fluorescence intensity (MFI) in parenthesis (*x*-axis). (**B**) Bar graphs extend from the 25^th^ to 75^th^ percentiles; the line in the middle represents the median of the pooled data. The whiskers delineate the smallest to the largest value. The data represent 2 individual experiments with 2 replicates in each experiment. Horizontal lines represent significant differences (*P*<0.05) between the indicated culture conditions.

Because after PA, PB and ST exposure we observed substantial increases in IL-8 in the absence of immune cells, we next measured IL-8 expression in supernatants from the organotypic models built with whole (total) or Mϕ-depleted PBMC. After 4 hours of exposure to either PA, PB, or ST, the tissues were disaggregated and cells used to measure IL-8 intracellular staining by flow cytometric analysis. We confirmed the epithelial cell expression of IL-8 and its independence from the presence of Mϕ (**Fig 11**). Regardless of the presence of Mϕ in organotypic models, comparable increases in the levels of expression of IL-8 were observed when exposed to *Salmonella* over those observed in media control cultures (**Fig 11**). Because in **Fig 3**we found that PA and PB induced higher secretion of IL-8 in the supernatants of organotypic models containing whole PBMC, as compared to supernatants of organotypic models built without PBMC, it is reasonable to speculate that cytokines secreted by immune cells modulate IL-8 secretion during inflammatory responses to PA and PB infections. This hypothesis is supported by previous studies showing that signals from lymphocytes are required for epithelial function [55–57].

### Gene expression following exposure to PA, PB, and ST

**Fig 12:**
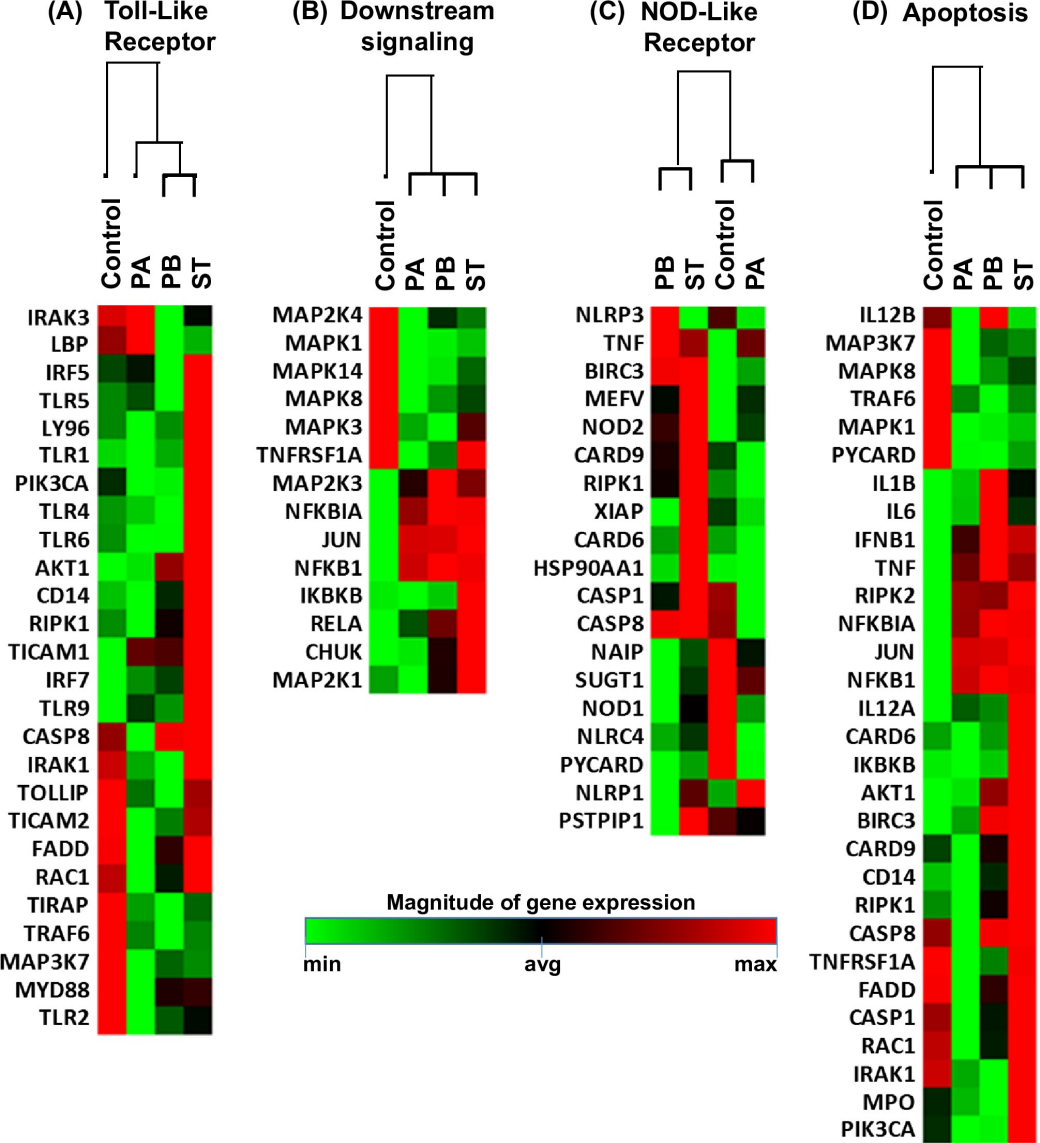
Expression of antimicrobial genes following stimulation of 3-D models with different *Salmonella* strains. 3-D organotypic models were exposed, or not, to either *Salmonella enterica* serovar Paratyphi A (PA), Paratyphi B (PB), or Typhi (ST) strains. After 4 hours, constructs were collected and used to measure gene expression by qRT-PCR. Data are shown as unsupervised clustergrams displaying hierarchical clustering of the dataset as a heat map. Geometric mean values of 2 independent experiments, each with 2 replicates are reported. Genes detected by the Antimicrobial Responses RT2 Profiler PCR Array. (**A**) Toll-Like Receptor (TLR) signaling. (**B**) Downstream signaling of antibacterial responses. (**C**) NOD-Like Receptor (NLR) signaling. (**D**) Apoptosis signaling.

**Fig 13:**
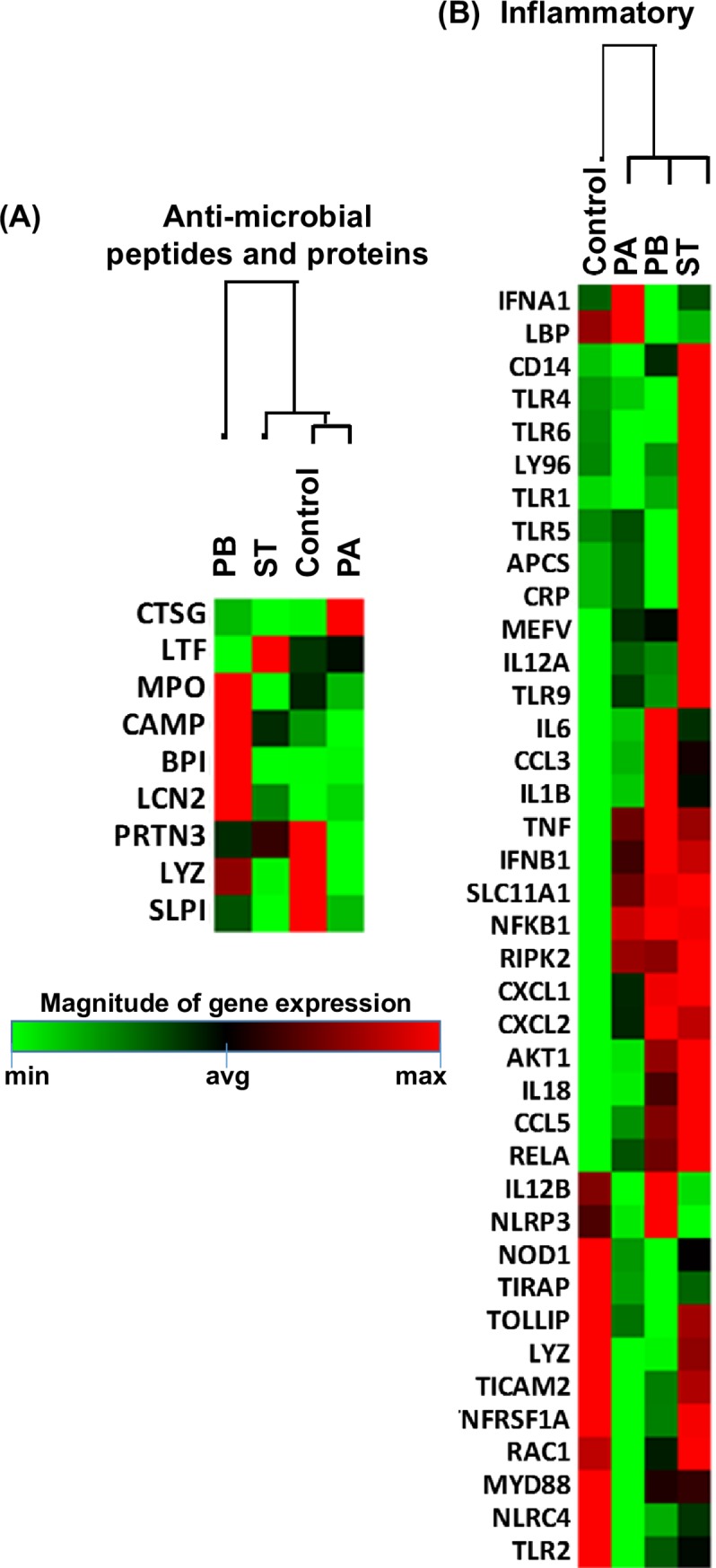
Anti-inflammatory responses after stimulation of 3-D organotypic models with different *Salmonella* strains. 3-D organotypic models were exposed or not to either *Salmonella enterica* serovar Paratyphi A (PA), Paratyphi B (PB), or Typhi (ST) strains. After 4 hours, constructs were collected and used to measure gene expression by qRT-PCR. Data are shown as unsupervised clustergrams displaying hierarchical clustering of the dataset as a heat map. Geometric mean values of 2 independent experiments, each with 2 replicates are reported. Genes detected by the Antimicrobial Responses RT2 Profiler PCR Array: (**A**) inflammatory and (**B**) anti-microbial peptide and protein signaling.

It is well known that ligand activation of cytokine/chemokine receptors stimulates several pathways, including Toll-like receptor (TLR) and downstream signaling. Thus, we next investigated whether the differential responses described above could be the result of defined antibacterial gene signatures. A set of ~84 genes, including those responsible for TLR and downstream signaling of antibacterial responses, as well as the NOD-like receptor (NLR), apoptosis, inflammatory, and anti-microbial peptide and protein signaling, were detected by the Anti-microbial Responses RT2 Profiler PCR Array. After 4 hours of infection, the organotypic models were collected and used to measure gene expression by qRT-PCR. We performed unsupervised clustergrams displaying the hierarchical clustering of the dataset as a heat map, with dendrograms indicating co-regulated genes. Surprisingly, the clustering of the genes showed similar antibacterial signatures between PB and ST, whilst the signatures following exposure to PA exhibited unique patterns (**Figs 12 and 13**). Of note, temporal differences between gene expression and the release of cytokines into culture supernatants [58, 59] might have been responsible for the lack of a tight concordance between these two measurements. Despite these temporal differences, gene clustering confirmed that the similarities and differences between PA, PB, and ST are related to the activation of different pathways that are *Salmonella* strain dependent.

## Discussion

In the present studies, we demonstrated that by changing the conditions of the crosstalk between immune cells, it is possible to modulate both the production of pro-inflammatory cytokines and the recruitment of inflammatory cells (*e*.*g*., Mϕ, PMNs) to the site of inflammation. Previous studies have described the critical role of Mϕ in *Salmonella* infections [60, 61] but the data presented here are, to the best of our knowledge, the first demonstration of a direct role of epithelial cells, lymphocytes, Mϕ and PMN crosstalk in controlling their function during PA, PB and ST infections.

Based on our observations, we postulate that innate cells alter the function of neighboring cells (including epithelial cells) in the gut microenvironment and that this phenomenon differs depending on individual *Salmonella* serovars. It is likely that when gut-resident Mϕ fail to contain the *Salmonella* infection they release cytokines/chemokines (*e*.*g*., IL-6, IL-8, TNF-α, and CCL3) to attract more macrophages and PMN to the diseased area. Of note, several of these cytokines/chemokines are likely to be co-regulated since they are secreted simultaneously. It is well known that insults to the intestinal mucosa, including those resulting from bacterial infections, promote Mϕ and PMN infiltration to the affected site [62–64]. To cross the epithelium, PMN might cause transient increases in the epithelial barrier permeability by breaking junctional complexes, which the PMN actively reseal after transmigration [65, 66]. Activated PMN will then engulf the bacteria, and, together with macrophages and epithelial cells, will mount a local bacterial defense and clearance response that will differ in function of the bacterial strain causing the insult. This rationale is consistent with our previous results employing a 3-D organotypic model similar to the one used in the present studies showing that different *S*. Typhi strains exhibiting high degrees of homology but with small variations in gene expression elicited dissimilar innate cell responses in the human intestinal mucosa [37], that may shape adaptive immune responses [32].

Since we observed that PB infection had a higher cytolytic effect on Mϕ compared to PA or ST infections, we propose that resident Mϕ infected with PB will die rapidly and be removed by Mϕ recruited to the site of inflammation. Upon clearance, the release of pro-inflammatory cytokines, such as IL-6, IL-8, and TNF-α, will decrease, reducing Mϕ trafficking to the site of infection without altering PMN migration. As shown by our blocking experiments, while neutralization of IL-6, IL-8, and TNF-α inhibited migration of Mϕ exposed to ST, it did not affect the migratory activity of PMN exposed to either PB or PA. We also observed that exposure of the PMN to supernatants from the PA and PB infected organotypic models built with whole PBMC exhibit significant reductions in PMN migration (**Fig 9B**) compared to the organotypic models built with Mϕ-depleted PBMC, indicating a major role of Mϕ in modulating PMN migration into the site of inflammation. Of note, we also found a direct correlation between IL-6 and TNF-α production. Current evidence suggests that TNF-α can both, depending on the experimental conditions, enhance or suppress the production of IL-6, a cytokine known to control PMN migration [67]. The results presented above are also supported by previous studies showing in a model of liver injury that a decline in IL-6 and TNF-α secretion is associated with PMN infiltration [68].

We also propose that Mϕ differentially increase their production of antimicrobial products (*e*.*g*., elastase) and regulate PMN function upon exposure to *Salmonella*. This proposition is in agreement with previous studies showing that dying infected cells decorated with antimicrobial proteins, such as elastase, regulate Mϕ functions such as the production of TNF-α [52, 69]. Thus, it is reasonable to speculate that while PA-infected Mϕ will decrease the cytokines/chemokines in the milieu; remaining viable Mϕ and PMN are likely to produce high amounts of CCL3, and perhaps other cytokines/chemokines, which will result in decreased migration of Mϕ but enhanced PMN migration. These assumptions are based on the PA major cytotoxic effect on Mϕ, and the data from the migration experiments. However, this phenomenon might not be as pronounced during ST infection, since, as for PB, ST did not elicit significant increases in the release of CCL3 into supernatants of models built with whole PBMC as compared with models built with Mϕ-depleted PBMC, suggesting that PA *vs*. PB & ST differ in their ability to evade the PMN-dependent host defense mechanism. Indeed, previous studies have shown that individuals infected with *S*. Typhi exhibited ileal mucosal hypertrophy caused primarily by Mϕ with PMN-poor exudates [70–73]. In this regard, one cannot exclude that the virulence-associated (Vi) capsular polysaccharide of ST as an impeding factor for bacterial-guided PMN chemotaxis. It has been shown that Vi of *S*. Typhi obstructs bacterial-guided PMN chemotaxis [74] by averting the phagocyte respiratory burst through inhibition of antibody-dependent complement activation [75]. Although PA, PB, and ST share a considerable genetic similarity, unlike ST, the Vi-antigen is not expressed by PA or PB strains [76]. Nonetheless, since the bacterial infection and PMN migration assays were performed in the absence of serum (*e*.*g*., plain RPMI) to avoid complement activation and opsonization, cytokines/chemokines are likely to have played a pivotal role in the PMN migration observed in our studies. Indeed, as shown by our blocking experiments, PMN migration was more sensitive to the neutralization of cytokines after ST infection than after PA and PB infections. Chemokines such as CCL3 serve as PMN attractors to the site of infection [42], and have protective roles in *Salmonella* infections [42, 77]. Thus, the differential oxidative environment and the antimicrobial responses (i.e., cytokines, and pathways activated following engagement of their receptors) produced in response to *Salmonella* infection are all likely to enable differential host immune responses elicited by the various *Salmonella* serovars [78].

To our knowledge, this is the first study to directly examine the role of lymphocytes, Mϕ, PMN and epithelial cell crosstalk in the control of their function (*i*.*e*., production of cytokine/chemokines and migration behavior) during infection with pathogens with high degrees of homology, such PA, PB and ST serovars. However, it should be noted that to simplify the experiments, only the heterogeneous preparation of lymphocytes, Mϕ, and PMN were used in this study. Thus, the role of cell subtypes (*e*.*g*., M1 and M2 Mϕ) in the differential chemotaxis observed in this study remains to be explored. Another important consideration is that it is very likely that in addition to IL-6, IL-8, TNF-α, and CCL3, other cytokines/chemokines also play important roles in differentially regulating chemotaxis in the mucosal microenvironment. Future investigations including a larger panel of cytokines and chemokines and the role of various cell subsets are needed to fully elucidate the rules governing the crosstalk among various cell types following infections with ST, PA, and PB. Finally, it is not possible to exclude that some of the observations and differences highlighted in this study were specific to the individual strains studied in this manuscript. For example, different *Salmonella* strains with different levels of Vi expression, different virulence, or different lengths of the LPS chains might trigger different host responses. This hypothesis is supported to some extent by the results described in **S2 Fig** using the wild-type ST strain Ty2 and 2 attenuated ST strains, Ty21a, and CVD 915. Each of attenuated ST strains was derived from the parent strain Ty2 but possess small variations in gene expression (*e*.*g*., Ty21a mutations in multiple genes, including *galE*, *galT*, *alK*, *galP*, *rpoS*, *ilvD*, *rcsC*, *tviC*, *tviE* and *vexD* [79], and CVD 915 with deletions in *guaBA*, interrupting the guanine biosynthesis pathway [80]). Different levels of Mϕ and neutrophil migration were observed among these different strains of ST.

In sum, our data strongly suggest that PA, PB, and ST affect the early innate immune responses in the gut differently. We also show the importance of crosstalk among lymphocytes, Mϕ, PMNs, and epithelial cells, which is cytokine/chemokine-dependent, bacteria serotype-specific, and play a pivotal role in orchestrating the mucosal innate, and likely adaptive, host inflammatory immune response in the mucosal microenvironment.

## Methods

### Ethics statement

Blood samples were collected from volunteers who gave informed, signed consent for participation in the University of Maryland Institutional Review Board approved protocol authorizing the collection of blood specimens from healthy adult blood donors for in vitro studies (HP-00040025).

### 3-D organotypic model of the human intestinal mucosa

The 3-D model setup and cultivation were performed as previously described [34–36, 38]. Briefly, a multi-step process is required to build the model. First, primary endothelial cells (HUVEC cells, CRL-1459, ATCC, Manassas, VA), primary fibroblasts (CCD-18Co cells, CRL-1459, ATCC), and HCT-8 epithelial cell line (CCL-244, ATCC) were grown as a confluent monolayer in order to reach enough cells to construct the model. The second step consisted of preparing an extracellular matrix (ECM) composed mainly of collagen-I enriched with with other gut basement membrane proteins (*i*.*e*., 10 μg/mL laminin (Sigma), 40 μg/mL collagen-IV (Sigma), 10 μg/mL fibronectin (BD Biosciences, San Jose, California, USA), 2 μg/mL heparin sulfate proteoglycan (Sigma). Then, fibroblasts and endothelial cells were embedded in the ECM, and the cell embedded-collagen transferred to the Rotating Wall Vessel (RWV) (Synthecon, Inc., Houston, Texas, USA) bioreactor containing ~10^7^ epithelial cells, and incubated at 37°C with 5% CO_2_. After 6 ± 1 and 13 ± 1 days, peripheral blood mononuclear cell (PBMC) were added to the system (~2 x 10^7^ cells/vessel). For “gain and loss” studies to define the importance of immune cells (i.e., lymphocytes and macrophages), models were set-up with or without PBMC. For “gain and loss” studies to define the importance of macrophages, these cells were removed from the PBMC by adhesion-mediated purification, for which PBMC were incubated for 2 hours on 2% gelatin-coated tissue culture flasks [47, 48]. As for conventional assays, for “gain and loss” studies, whole or macrophage-depleted PBMCs were added into RWV vessels on days 6 ± 1 and 13 ± 1 after model set up.

### Bacterial growth

Isolates of wild-type PA strains CV223 (ATCC#9150) [25, 26] and 01–0020 [81], PB strains CV23 (clinical isolate from Chile)[25, 26] and 02–0303 [81], and ST strains Ty2 [37], and ISP1820 [34] and typhoid vaccine Ty21a [37, 79] were streaked onto Luria-Bertani (LB) agar Lennox (Difco Laboratories, Detroit, MI) plates and allowed to incubate at 37°C and 5% CO_2_. Attenuated vaccine candidate ST strains CVD 915 [37, 80] and CVD 908 [37, 82] were grown on solid medium supplemented 1% Guanine or 0.1% 2, 3-Dihydroxybenzoic acid (DHB) (Sigma, St. Louis, MO), respectively. After overnight incubation, each strain suspension was diluted to obtain an optical density (OD) value of 0.2, corresponding to a suspension of ~10^8^ bacterial cells per mL [83]. Quantification of each strain was performed by λ-600 OD spectrophotometric analysis. Unless otherwise stated, experiments were performed using PA strain CV223, PB strain CV23, and ST strain Ty2.

### Infection of the 3-D organotypic model with *Salmonella* PA, PB, and ST

Infection of the 3-D model was performed as previously described [34, 36]. Briefly, the vessels were removed from the RWV bioreactor and the constructs within were washed twice with RPMI to remove any residual of antibiotic and non-resident cells. The vessels were then refilled with RPMI. Except for the negative controls (media only), the appropriate bacterial suspension (approximately 100 multiplicity of infection (MOI)) was then added to all vessels. The vessels were then returned to the RWV bioreactor and incubated at 37°C and 5% CO_2_ for up to 6 hours before the experiment was terminated, and supernatants and constructs were collected from each vessel for further analysis.

### Elastase, myeloperoxidase and cytokine production

Levels of elastase and myeloperoxidase (MPO) in culture supernatants were measured by using commercial NETosis and PMN Activity Myeloperoxidase Assay kits, respectively (Cayman Chemical, Ann Arbor, MI). The NETosis Assay kit employs a specific chromogenic substrate (N-methoxysuccinyl-Ala-Ala-Pro-Val p-nitroanilide), which is selectively cleaved by elastase. The rate of enzymatic hydrolysis of the substrate is followed by the increase in absorbance due to the release of 4-nitroaniline. The PMN Activity Myeloperoxidase Assay kit utilizes 3,3’,5,5’-tetramethyl-benzidine (TMB) as a chromogenic substrate, which in the presence of MPO yields a blue color detectable by spectrophotometer. Levels of interleukin (IL)-1β, IL-6, IL-8, tumor necrosis factor (TNF)-α and Chemokine (C-C motif) ligand 3 (CCL3), also known as macrophage inflammatory protein 1-alpha (MIP-1α), were measured by using the Meso Scale Discovery (MSD, Gaithersburg, MD) multiplexed-assay. Supernatants were harvested 4 hours after *Salmonella* infection and kept at -20°C until assayed. In these studies, uninfected cells (medium only) were used as controls. ELISA and MSD assays were carried out following the manufacturer’s instructions. The level of sensitivity of elastase and MPO ELISAs were 0.2 mU/ml and 28 ng/ml, respectively. The levels of sensitivity for the various cytokines measured by MSD ranged from 0.3–2.5 pg/ml

### Cell viability quantified by lactate dehydrogenase (LDH)

The viability of the cells was assessed by quantifying the LDH release into the supernatant using a commercial kit (CytoTox 96; Promega, Madison, WI) as previously described [37]. Briefly, supernatants were harvested 4 hours after exposure to the different serovars and kept at -20°C until assayed. The LDH Positive Control was used to create a standard curve and interpolate the sample results to obtain the relative number of lysed cells.

### Isolation of PMN from blood

PMN were isolated as previously described with a few modifications [84]. Briefly, blood collected from healthy volunteers were diluted 1:3 with 1x Phosphate Buffer Solution (PBS) and layered, up to 35 mL, over 15 mL of Ficoll-Paque density gradient media. This mixture was centrifuged for 30 minutes at 25°C allowing the formation of a denser precipitate of erythrocytes with overlying buffy coat of PMNs. After centrifugation, the upper layers (e.g., plasma, PBMC, and Ficoll-Paque) were removed, and the remaining layer containing erythrocytes and PMNs resuspended in 1x PBS to the original blood volume before adding an equal volume of 6% Dextran-500 solution. After homogenization by inversion, the tubes were allowed to sediment. After 1 hour, the leukocyte-rich, erythrocyte-poor supernatant was aspirated and transferred into another 50mL conical tube. To lyse the remaining erythrocytes, cells were centrifuged at 25°C, the supernatant discarded, and the pelleted cells resuspended in 12 mL of ice-cold ddH_2_O for 20 seconds before adding 1.2 mL of 10X PBS and diluting up to 50 mL with 1X PBS. This step was repeated a second time if needed. The purity of the cells was confirmed by flow cytometry by gating them based on their light scatter characteristics and specific lineage differentiation markers: CD11b+, CD11c+, CD14-, CD19-, CD45+. Analyses were performed in an LSR II flow cytometer (BD Biosciences) in the UMB Flow Cytometry and Mass Cytometry Core. PMN populations were >90% pure.

### Macrophage & PMN migration experiments

Macrophages (Mϕ) were generated from the human monocyte cell line U937 (CRL-1593.2, ATCC) using a phorbol 12-myristate 13-acetate (PMA) protocol [49]. Briefly, U937 monocytes were incubated with 6.25 ng/ml [10nM] of PMA in RPMI 1640 (Gibco, Grand Island, New York) media supplemented with 100 U/mL penicillin, 100 μg/mL streptomycin, 50 μg/mL gentamicin, 2 mM L-glutamine, 1 mM sodium pyruvate, 10 mM HEPES (Gibco) buffer and 10% heat-inactivated fetal bovine (FBS) serum (R10). After 48 hours, culture flasks were washed twice with plain RPMI, and cells allowed to differentiate for another 24 hs in R10 media.

Human PMN were isolated, as described above. Both PMN and macrophage migration experiments were performed using previously described protocols [53]. Briefly, a mixture was prepared by combining 800 μL of 5 mg/ml bovine collagen-I with 100 μL of 10x DMEM (Invitrogen, Camarillo, CA, USA). Next, NaOH was added to the mixture to attain neutral pH, as assessed by a phenol red color change. The mixture was then diluted with 1X Hanks’ buffered saline solution (HBSS) supplemented with 2% FCS and 10 mM HEPES to a final concentration of 4.8 mg/mL. To establish an extracellular matrix that could facilitate PMN/macrophage anchoring and chemoattractant gradient formation, 70 μL of this collagen mixture was deposited into the well inserts of a 24-well plate (8 μm pore)(Corning, NY, USA) and allowed to gelify for 1 hour at 37°C and 5% CO_2_. After gelification was completed, the wells were filled, in triplicate, with 300 μL of supernatant from the appropriate RWV infection experiment diluted 1:3 with RPMI. For PMN migration assays, positive control conditions consisted of 300 μL of a 4 ng/mL solution of N-formyl-methionine-leucine-phenylalanine (n-formyl-MLF) prepared in buffer A (PBS supplemented with 2% FCS and 10mM HEPES). For macrophage migration assays, positive control conditions consisted of 300 μL of a 100 ng/mL solution of CCL3 prepared in buffer A. Matrix-laden well inserts were then allowed to bathe in underlying supernatants for a 2 hour incubation period to facilitate gradient formation before buffer A was added to each insert and pre-warmed to 37°C in the incubator. Thus, 25 μL of a 1 x 10^7^ cells/mL suspension of isolated PMNs or macrophages (~2.5 x 10^5^ cells) was added into each well-insert and incubated for a 3 (PMNs) or 4 hours (macrophages) at 37°C and 5% CO_2_. After incubation, the insert was removed, and PMN and macrophage migrations were visualized using a Nikon Eclipse TE2000-S inverted microscope (Nikon, Melville, NY, USA) under bright field setting and 10x objective lens. NIS-Elements BR software (Nikon) was used to take photographs, and quantification of cell migration was performed using ImageJ software (NIH).

### Antibodies and fluorochromes

Cells were surface stained with anti-human monoclonal antibodies (mAbs) to CD11b (clone ICRF44), CD11c (clone B-ly6), CD14 (clone TuK4), CD45 (clone 2D1), CD66c (clone B6.2/CD66), CD163 (clone GHI/61), IL-6 (clone MQ2-39C3), TNF-α (clone MAb11), (BD Pharmingen, San Diego, CA), CD19 (clone SJ25-C1), CCL3 (clone CR3M)(Invitrogen, Carlsbad, CA), IL-8 (clone E8N1), and CD326 (EpCAM, clone 9C4) (Biolegend, San Diego, CA). These mAbs were directly conjugated to the following fluorochromes: Fluorescein isothiocyanate (FITC), Phycoerythrin (PE), Peridinin chlorophyll protein (PerCP)-Cy5.5, PE-Cy7, Energy Coupled Dye PE-Texas-Red conjugate (ECD), Pacific Blue, Brilliant Violet (BV) 570, BV605, BV650, Quantum dot (QD) 800, Alexa 647, allophycocyanin (APC)-Alexa 700, or APC-H7.

### 3-D model construct staining and flow cytometry analysis

After 4 hours of exposure to *S*. Typhi, PA or PB strains, the constructs were harvested and used to isolate cells by a 2-hour incubation with collagenase enzyme and additional mechanical agitation. Briefly, constructs were covered with 10 mg/ml (1%) of collagen/dispase (Roche, Indianapolis, IN) and vigorously re-suspended up and down with a transfer pipette. After a 30 minute incubation in a 37°C 5% CO2 incubator, an 18-G needle fitted on a 5-ml syringe was used to further disrupt the construct by passing the collagenase solution through the needle 3 times and then returning the tube to the 37^°^C 5% CO2 incubator. After an additional 30 minutes, the pieces were again vigorously resuspended up and down with a transfer pipette and filtered through a 40 μm filter to obtain single cells.

For flow cytometric assays, single cells were stained with a dead-cell discriminator, violet fluorescent viability dye (ViViD, Invitrogen)[83], followed by the blocking of Fc-receptors with purified human IgG, surface staining and fixation and permeabilization with Fix & Perm cell buffers (Invitrogen, Carlsbad, CA)[83]. Cells were then stained intracellularly for IL-6, IL-8, CCL3, and TNF-α, and fixed with 1% formaldehyde. Data were analyzed by flow cytometry on an LSR-II instrument (BD Biosciences) and WinList v9.0 (Verity Software House, Topsham, ME). Cells were gated based on their light scatter characteristics and specific lineage differentiation markers: CD45+CD163-CD66c+ for PMN, CD45+CD14+CD163+CD11b+ for macrophages, and CD45- EpCAM+ for epithelial cells. Flow cytometry experiments were performed at the Flow Cytometry and Mass Cytometry Core Facility of the University of Maryland School of Medicine Center for Innovative Biomedical Resources (CIBR), Baltimore, Maryland.

### Human antibacterial RT2 profiler arrays

Isolation of total cellular RNA was performed as previously described [36]. Briefly, total RNA was extracted using RNeasy Mini Kits (Qiagen). The RNA samples were then converted to cDNA and subjected to qPCR amplification using the QuantiTect SYBR Green Kit (Qiagen) on an ABI 7900HT Fast Real-Time PCR System ((Applied Biosystems, Foster City, CA). Analyses of results were performed using the web-based GeneGlobe Data Analysis Center web-based Software (Qiagen)(https://www.qiagen.com/us/shop/genes-and-pathways/data-analysis-center-overview-page/). The software automatically selected an optimal set of internal control/housekeeping/ normalization genes for the analysis from the available housekeeping gene panel (i.e., ACTB, B2M, GAPDH, HPRT1, and RPLP0) on the PCR Array. The CT values for these genes were then geometrically averaged and used for the 2(−Delta Delta Ct) calculations. The software also performs unsupervised clustergram displaying hierarchical clustering of the dataset as a heat map with dendrograms indicating co-regulated genes. Clustergrams are based on hierarchical clustering method that (1) assigns each gene to its own cluster (agglomerative), (2) joins the nearest clusters, and (3) re-estimate the distance between clusters. Experimental variables such as treatment do not guide or bias cluster building. To create a hierarchical cluster, the magnitude of gene expression is determined by calculating the 2^–ΔCT^ for each individual gene and normalizing to the average 2^–ΔCT^ of all genes across all arrays. A set of 84 genes was profiled including those responsible for Toll-Like Receptor (TLR) signaling (*e*.*g*., TLR1, TLR4, TLR5 and TLR9), downstream signaling of antibacterial responses (*e*.*g*., Nfκb1, NFKBIA, MAP2K1, and Jun), NOD-Like Receptor (NLR) signaling (*e*.*g*., NIrp C4, NIrp1a, NIrp3, NOD1 and NOD2), apoptosis signaling (*e*.*g*., Card6, CASP1, and CASP8), anti-microbial peptides and proteins (*e*.*g*., Mop, Prtn3, Lyz, and Ltf), and inflammation (*e*.*g*., IL-6, IL-1b, CCL3, and Myd88).

### Statistical analysis

All statistical tests were performed using Prism software (version 6.0, GraphPad Software, La Jolla, CA). Comparisons between groups were performed using One-way ANOVA, with Geisser-Greenhouse corrections, with individual variances computed for each comparison. Correlations used the Pearson Product Moment tests. P values <0.05 were considered significant.

## Supporting information

S1 Fig Macrophage viability and cytokine production after stimulation with different *Salmonella* serovars. 3-D organotypic models built with whole (Total) PBMC were exposed or not to either *Salmonella enterica* serovar Paratyphi A (PA, strains 01–0020 and CV223), Paratyphi B (PB, strains 02–0303 and CV23), or Typhi (ST, strains Ty2, ISP1820 or CVD 908). After 4 hours, supernatants were collected and used to stimulate macrophages. Macrophages were obtained as in Fig 6. After 3 hours of incubation, macrophages were harvested and used either (**A**) to measure cell viability by using the trypan blue exclusion test, or (**B-F**) to measure cytokine expression. (**B**) Overview of the controls (unstained and unstimulated [media only] cells), as well as a representative experimental condition (i.e., PA 010020) to evaluate the levels of IL-6, IL-8, TNF-α and CCL3 intracellular cytokines by flow cytometry. Bars representing mean ± SE of one independent experiment with 3 replicates are shown for IL-6 (**C**), IL-8 (**D**), TNF-α (**E**), and CCL3 (**F**). Horizontal lines represent significant differences (*P*<0.05) between the indicated culture conditions. Complete list of *P* values is shown in **S3 Table**.
(PDF)

S2 Fig Macrophage and neutrophil migration following exposure to different *Salmonella* serovars. 3-D organotypic models built with whole (Total) PBMC were exposed or not to either *Salmonella enterica* serovar Paratyphi A (PA, strain 01–0020), Paratyphi B (PB, strain 02–0303), or Typhi (ST, strains Ty2, Ty21a or CVD 915). After 4 hours, supernatants were collected and used to stimulate (**A**) macrophage and (**B**) neutrophil migration in a trans-well system. Macrophages and neutrophils were obtained as in Figs 6 & 9, respectively. Bar graphs extend from the 25^th^ to 75^th^ percentiles; the line in the middle represents the median of the pooled data. The whiskers delineate the smallest to the largest value. The data represent one of two individual experiments, each experiment with 5 replicates. Horizontal lines represent significant differences (*P*<0.05) between the indicated culture conditions.
(PDF)

S1 Table Statistical analyses of Fig 3. (DOCX)

S2 Table Statistical analyses of Fig 4C. (DOCX)

S3 Table Statistical analyses of S1 Fig. (DOCX)

S4 Table Statistical analyses of Fig 6B. (PDF)

S5 Table Statistical analyses of Fig 7. (PDF)

S6 Table Statistical analyses of Fig 8A. (PDF)

S7 Table Statistical analyses of Fig 9B. (PDF)
