# Supplementary material for: Crosstalk between leukocytes triggers differential immune responses against Salmonella enterica serovars Typhi and Paratyphi
Source: PLoS Negl Trop Dis. 2019 Aug 14;13(8):e0007650. doi: 10.1371/journal.pntd.0007650 (PMC6709971; doi:10.1371/journal.pntd.0007650)
Supplement: S1 Fig — 3-D organotypic models built with whole (Total) PBMC were exposed or not to either Salmonella enterica serovar Paratyphi A (PA, strains 01–0020 and CV223), Paratyphi B (PB, strains 02–0303 and CV23), or Typhi (ST, strains Ty2, ISP1820 or CVD 908). After 4 hours, supernatants were collected and used to stimulate macrophages. Macrophages were obtained as in Fig 6. After 3 hours of incubation, macrophages were harvested and used either (A) to measure cell viability by using the trypan blue exclusion test, or (B-F) to measure cytokine expression. (B) Overview of the controls (unstained and unstimulated [media only] cells), as well as a representative experimental condition (i.e., PA 010020) to evaluate the levels of IL-6, IL-8, TNF-α and CCL3 intracellular cytokines by flow cytometry. Bars representing mean ± SE of one independent experiment with 3 replicates are shown for IL-6 (C), IL-8 (D), TNF-α (E), and CCL3 (F). Horizontal lines represent significant differences (P<0.05) between the indicated culture conditions. Complete list of P values is shown in S3 Table. (PDF) [file pntd.0007650.s001.pdf]

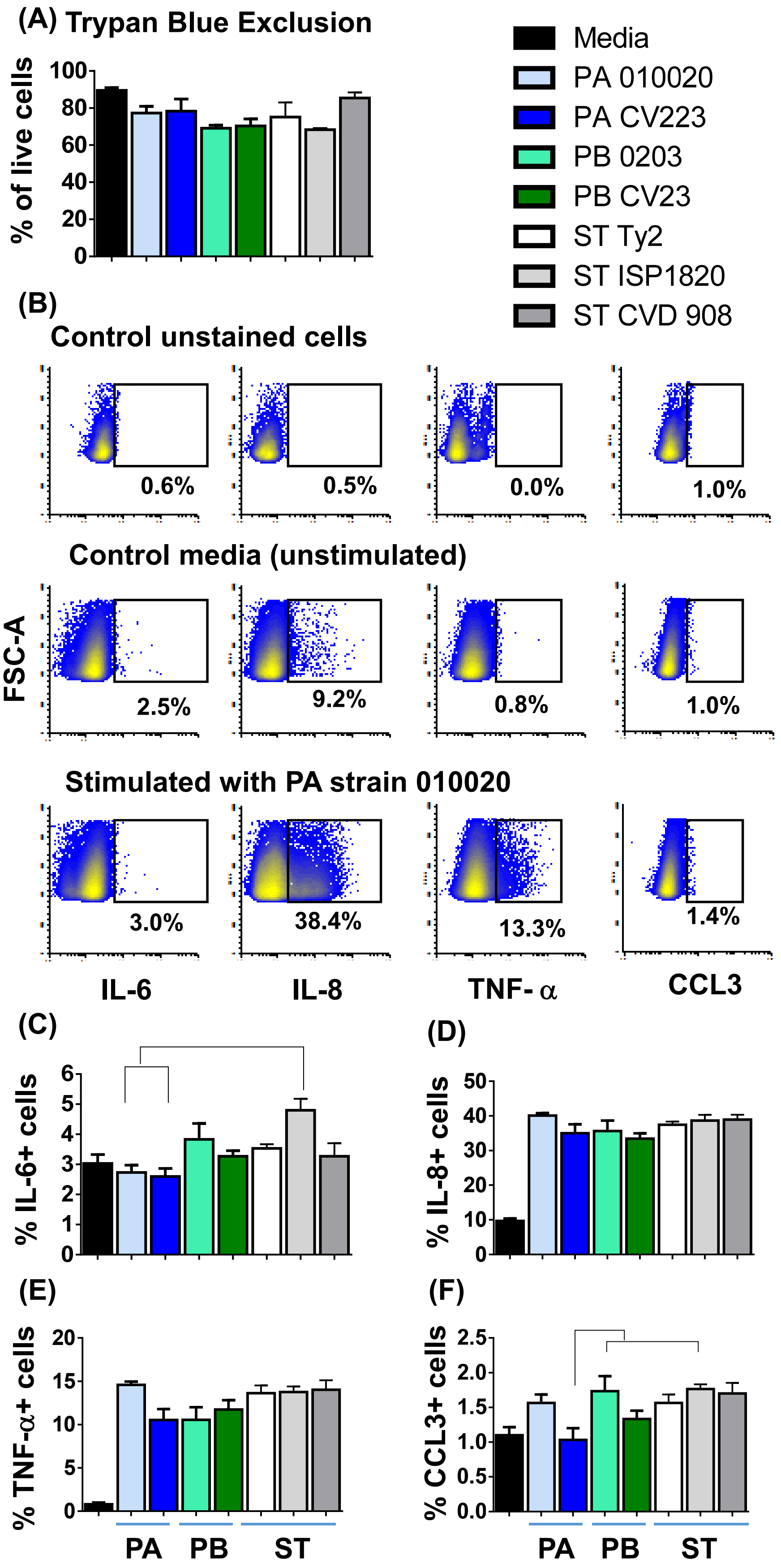
(A) Trypan Blue Exclusion
% of live cells
100
80
60
40
20
0
Media
PA 010020
PA CV223
PB 0203
PB CV23
ST Ty2
ST ISP1820
ST CVD 908
(B)
Control unstained cells
0.6%
0.5%
0.0%
1.0%
Control media (unstimulated)
FSC-A
2.5%
9.2%
0.8%
1.0%
Stimulated with PA strain 010020
3.0%
38.4%
13.3%
1.4%
IL-6
IL-8
TNF- α
CCL3
(C)
% IL-6+ cells
6
5
4
3
2
1
0
(D)
% IL-8+ cells
50
40
30
20
10
0
(E)
% TNF-α+ cells
20
15
10
5
0
PA
PB
ST
(F)
% CCL3+ cells
2.5
2.0
1.5
1.0
0.5
0.0
PA
PB
ST
