# Supplementary figures and images for: Crosstalk between leukocytes triggers differential immune responses against Salmonella enterica serovars Typhi and Paratyphi
Source: PLoS Negl Trop Dis. 2019 Aug 14;13(8):e0007650. doi: 10.1371/journal.pntd.0007650 (PMC6709971; doi:10.1371/journal.pntd.0007650)

**(A) Macrophages**

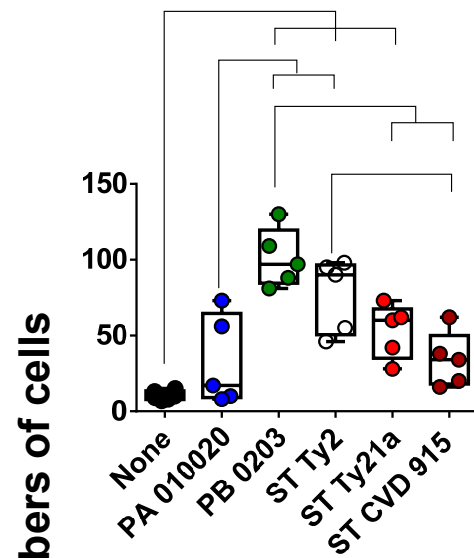

**(B) Neutrophils**

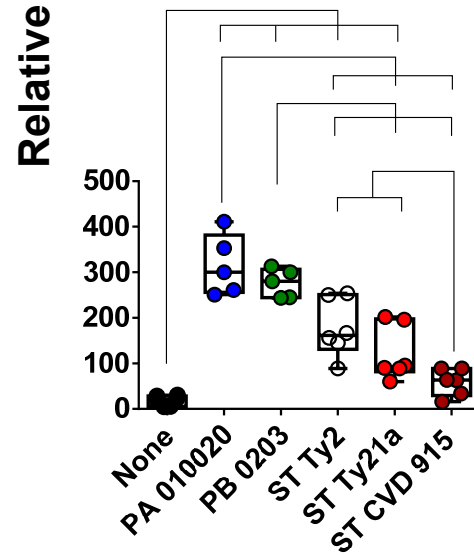

***Salmonella enterica* serovars**

Supplement: S2 Fig — 3-D organotypic models built with whole (Total) PBMC were exposed or not to either Salmonella enterica serovar Paratyphi A (PA, strain 01–0020), Paratyphi B (PB, strain 02–0303), or Typhi (ST, strains Ty2, Ty21a or CVD 915). After 4 hours, supernatants were collected and used to stimulate (A) macrophage and (B) neutrophil migration in a trans-well system. Macrophages and neutrophils were obtained as in Figs 6 & 9, respectively. Bar graphs extend from the 25th to 75th percentiles; the line in the middle represents the median of the pooled data. The whiskers delineate the smallest to the largest value. The data represent one of two individual experiments, each experiment with 5 replicates. Horizontal lines represent significant differences (P<0.05) between the indicated culture conditions. (PDF) [file pntd.0007650.s002.pdf]
