# Supplementary material for: Crosstalk between leukocytes triggers differential immune responses against Salmonella enterica serovars Typhi and Paratyphi
Source: PLoS Negl Trop Dis. 2019 Aug 14;13(8):e0007650. doi: 10.1371/journal.pntd.0007650 (PMC6709971; doi:10.1371/journal.pntd.0007650)
Supplement: S4 Table — (PDF) [file pntd.0007650.s006.pdf]

S4 Table. Statistical analyses of **Fig 6B**

1way ANOVA	Significant?	Individual *P* Value
None 1[†] vs. PA 1	Yes	< 0.0001
None 1 vs. PB 1	Yes	< 0.0001
None 1 vs. ST 1	Yes	< 0.0001
None 1 vs. None 2[‡]	No	0.0666
None 1 vs. PA 2	Yes	< 0.0001
None 1 vs. PB 2	Yes	< 0.0001
None 1 vs. ST 2	Yes	< 0.0001
PA 1 vs. PB 1	Yes	< 0.0001
PA 1 vs. ST 1	Yes	< 0.0001
PA 1 vs. None 2	Yes	< 0.0001
PA 1 vs. PA 2	Yes	0.0001
PA 1 vs. PB 2	Yes	< 0.0001
PA 1 vs. ST 2	Yes	0.0307
PB 1 vs. ST 1	No	0.0651
PB 1 vs. None 2	Yes	< 0.0001
PB 1 vs. PA 2	Yes	0.034
PB 1 vs. PB 2	Yes	< 0.0001
PB 1 vs. ST 2	Yes	0.0001
ST 1 vs. None 2	Yes	< 0.0001
ST 1 vs. PA 2	No	0.9259
ST 1 vs. PB 2	Yes	< 0.0001
ST 1 vs. ST 2	Yes	< 0.0001
None 2 vs. PA 2	Yes	< 0.0001
None 2 vs. PB 2	Yes	< 0.0001
None 2 vs. ST 2	Yes	< 0.0001
PA 2 vs. PB 2	Yes	< 0.0001
PA 2 vs. ST 2	Yes	0.0002
PB 2 vs. ST 2	Yes	0.0036

†, culture with Mϕ-depleted PBMC

‡, culture with total PBMC
