# Supplementary material for: Crosstalk between leukocytes triggers differential immune responses against Salmonella enterica serovars Typhi and Paratyphi
Source: PLoS Negl Trop Dis. 2019 Aug 14;13(8):e0007650. doi: 10.1371/journal.pntd.0007650 (PMC6709971; doi:10.1371/journal.pntd.0007650)
Supplement: S5 Table — (PDF) [file pntd.0007650.s007.pdf]

S5 Table. Statistical analyses of **Fig 7**

	Elastase		Myeloperoxidase (MPO)	
1way ANOVA	Significant?	Individual *P* Value	Significant?	Individual *P* Value
None 1† vs. PA 1	Yes	< 0.0001	Yes	0.0001
None 1 vs. PB 1	No	0.1003	Yes	0.0065
None 1 vs. ST 1	Yes	0.0135	Yes	0.0425
None 1 vs. None 2‡	No	0.9636	No	0.1642
None 1 vs. PA 2	Yes	0.0004	Yes	0.0002
None 1 vs. PB 2	Yes	< 0.0001	Yes	< 0.0001
None 1 vs. ST 2	Yes	< 0.0001	Yes	< 0.0001
PA 1 vs. PB 1	Yes	0.0135	No	0.3901
PA 1 vs. ST 1	No	0.1003	Yes	0.0444
PA 1 vs. None 2	Yes	< 0.0001	Yes	< 0.0001
PA 1 vs. PA 2	No	0.2561	No	0.4985
PA 1 vs. PB 2	No	0.1602	No	0.4369
PA 1 vs. ST 2	No	0.891	No	0.981
PB 1 vs. ST 1	No	0.3857	No	0.3388
PB 1 vs. None 2	No	0.0647	Yes	< 0.0001
PB 1 vs. PA 2	No	0.0785	No	0.7278
PB 1 vs. PB 2	Yes	< 0.0001	No	0.108
PB 1 vs. ST 2	Yes	0.0068	No	0.3381
ST 1 vs. None 2	Yes	0.0053	Yes	0.0003
ST 1 vs. PA 2	No	0.4387	No	0.1128
ST 1 vs. PB 2	Yes	0.0014	Yes	0.0029
ST 1 vs. ST 2	No	0.0785	Yes	0.0236
None 2 vs. PA 2	Yes	< 0.0001	Yes	< 0.0001
None 2 vs. PB 2	Yes	< 0.0001	Yes	< 0.0001
None 2 vs. ST 2	Yes	< 0.0001	Yes	< 0.0001
PA 2 vs. PB 2	Yes	0.0025	No	0.0953
PA 2 vs. ST 2	No	0.2213	No	0.4187
PB 2 vs. ST 2	No	0.0608	No	0.3844

†, culture with Mϕ-depleted PBMC

‡, culture with total PBMC
