# Supplementary material for: Crosstalk between leukocytes triggers differential immune responses against Salmonella enterica serovars Typhi and Paratyphi
Source: PLoS Negl Trop Dis. 2019 Aug 14;13(8):e0007650. doi: 10.1371/journal.pntd.0007650 (PMC6709971; doi:10.1371/journal.pntd.0007650)
Supplement: S6 Table — (PDF) [file pntd.0007650.s008.pdf]

S6 Table. Statistical analyses of **Fig 8A**

1way ANOVA	Significant?	Individual *P* Value
None 1[†] vs. PA 1	Yes	0.0008
None 1 vs. PB 1	Yes	0.0008
None 1 vs. ST 1	Yes	0.0034
None 1 vs. None 2[‡]	No	0.0758
None 1 vs. PA 2	Yes	< 0.0001
None 1 vs. PB 2	Yes	< 0.0001
None 1 vs. ST 2	Yes	0.0004
PA 1 vs. PB 1	Yes	0.0008
PA 1 vs. ST 1	Yes	0.0066
PA 1 vs. None 2	Yes	0.0291
PA 1 vs. PA 2	Yes	0.011
PA 1 vs. PB 2	Yes	0.0002
PA 1 vs. ST 2	Yes	0.0011
PB 1 vs. ST 1	Yes	< 0.0001
PB 1 vs. None 2	Yes	< 0.0001
PB 1 vs. PA 2	Yes	0.0496
PB 1 vs. PB 2	No	0.93
PB 1 vs. ST 2	No	0.0709
ST 1 vs. None 2	Yes	0.0004
ST 1 vs. PA 2	No	0.4787
ST 1 vs. PB 2	No	0.0551
ST 1 vs. ST 2	No	0.4934
None 2 vs. PA 2	Yes	0.0128
None 2 vs. PB 2	Yes	0.0001
None 2 vs. ST 2	Yes	0.0002
PA 2 vs. PB 2	Yes	0.004
PA 2 vs. ST 2	No	0.1131
PB 2 vs. ST 2	Yes	0.0045

†, culture with Mϕ-depleted PBMC

‡, culture with total PBMC
