# Supplementary material for: Crosstalk between leukocytes triggers differential immune responses against Salmonella enterica serovars Typhi and Paratyphi
Source: PLoS Negl Trop Dis. 2019 Aug 14;13(8):e0007650. doi: 10.1371/journal.pntd.0007650 (PMC6709971; doi:10.1371/journal.pntd.0007650)
Supplement: S7 Table — (PDF) [file pntd.0007650.s009.pdf]

S7 Table. Statistical analyses of **Fig 9B**

1way ANOVA	Significant?	Individual *P* Value
None 1[†] vs. PA 1	Yes	0.0015
None 1 vs. PB 1	Yes	0.0005
None 1 vs. ST 1	Yes	0.0002
None 1 vs. None 2[‡]	No	0.7082
None 1 vs. PA 2	Yes	0.0015
None 1 vs. PB 2	Yes	0.0005
None 1 vs. ST 2	Yes	0.004
PA 1 vs. PB 1	No	0.0908
PA 1 vs. ST 1	Yes	0.0042
PA 1 vs. None 2	Yes	0.0016
PA 1 vs. PA 2	Yes	0.0027
PA 1 vs. PB 2	Yes	0.0021
PA 1 vs. ST 2	Yes	0.0068
PB 1 vs. ST 1	Yes	0.0007
PB 1 vs. None 2	Yes	0.0005
PB 1 vs. PA 2	Yes	0.0069
PB 1 vs. PB 2	Yes	0.0011
PB 1 vs. ST 2	Yes	0.0027
ST 1 vs. None 2	Yes	0.0004
ST 1 vs. PA 2	Yes	0.0121
ST 1 vs. PB 2	No	0.3757
ST 1 vs. ST 2	No	0.2957
None 2 vs. PA 2	Yes	0.0015
None 2 vs. PB 2	Yes	0.0006
None 2 vs. ST 2	Yes	0.0027
PA 2 vs. PB 2	Yes	0.0026
PA 2 vs. ST 2	Yes	0.0262
PB 2 vs. ST 2	No	0.6866

†, culture with Mϕ-depleted PBMC

‡, culture with total PBMC
