# Supplementary material for: Crosstalk between leukocytes triggers differential immune responses against Salmonella enterica serovars Typhi and Paratyphi
Source: PLoS Negl Trop Dis. 2019 Aug 14;13(8):e0007650. doi: 10.1371/journal.pntd.0007650 (PMC6709971; doi:10.1371/journal.pntd.0007650)
Supplement: Supplementary file 7 [file pntd.0007650.s003.docx]

S1 Table. Statistical analyses of **Fig 3**

1way ANOVA	IL-1β		IL-6		IL-8		TNF-α		CCL3	
	Significant?	Individual *P* Value	Significant?	Individual *P* Value	Significant?	Individual *P* Value	Significant?	Individual *P* Value	Significant?	Individual *P* Value
None 1[†] vs. PA 1	No	0.998	No	0.3184	Yes	0.0007	Yes	0.0005	No	0.6993
None 1 vs. PB 1	No	0.9928	No	0.2105	Yes	0.0004	Yes	< 0.0001	No	0.8979
None 1 vs. ST 1	No	0.9967	No	0.4318	Yes	< 0.0001	Yes	0.0015	No	0.4994
None 1 vs. None 2[‡]	No	0.9863	No	0.1063	No	0.813	No	0.4166	No	0.7196
None 1 vs. PA 2	Yes	0.0005	Yes	< 0.0001	Yes	< 0.0001	Yes	0.0066	Yes	< 0.0001
None 1 vs. PB 2	Yes	< 0.0001	Yes	< 0.0001	Yes	< 0.0001	Yes	0.0018	Yes	< 0.0001
None 1 vs. ST 2	No	0.0616	Yes	< 0.0001	Yes	< 0.0001	Yes	0.0137	Yes	< 0.0001
PA 1 vs. PB 1	No	0.9909	No	0.7899	No	0.7894	No	0.055	No	0.7961
PA 1 vs. ST 1	No	0.9948	No	0.8277	No	0.2794	Yes	0.0395	No	0.7703
PA 1 vs. None 2	No	0.9843	Yes	0.0131	Yes	0.0013	Yes	0.0003	No	0.4584
PA 1 vs. PA 2	Yes	0.0005	Yes	< 0.0001	Yes	0.0088	Yes	0.0084	Yes	< 0.0001
PA 1 vs. PB 2	Yes	< 0.0001	Yes	< 0.0001	Yes	0.0206	Yes	0.0024	Yes	< 0.0001
PA 1 vs. ST 2	No	0.0613	Yes	< 0.0001	Yes	0.0203	Yes	0.0235	Yes	< 0.0001
PB 1 vs. ST 1	No	0.9961	No	0.6291	No	0.4105	Yes	0.0219	No	0.583
PB 1 vs. None 2	No	0.9935	Yes	0.0071	Yes	0.0007	Yes	< 0.0001	No	0.6267
PB 1 vs. PA 2	Yes	0.0005	Yes	< 0.0001	Yes	0.016	Yes	0.0095	Yes	< 0.0001
PB 1 vs. PB 2	Yes	< 0.0001	Yes	< 0.0001	Yes	0.0364	Yes	0.0029	Yes	< 0.0001
PB 1 vs. ST 2	No	0.0627	Yes	< 0.0001	Yes	0.0361	Yes	0.0295	Yes	< 0.0001
ST 1 vs. None 2	No	0.9895	Yes	0.0213	Yes	< 0.0001	Yes	0.0011	No	0.3051
ST 1 vs. PA 2	Yes	0.0005	Yes	< 0.0001	No	0.0897	Yes	0.0075	Yes	< 0.0001
ST 1 vs. PB 2	Yes	< 0.0001	Yes	< 0.0001	No	0.1777	Yes	0.0019	Yes	< 0.0001
ST 1 vs. ST 2	No	0.0621	Yes	< 0.0001	No	0.1762	Yes	0.0194	Yes	< 0.0001
None 2 vs. PA 2	Yes	0.0006	Yes	< 0.0001	Yes	< 0.0001	Yes	0.0065	Yes	< 0.0001
None 2 vs. PB 2	Yes	< 0.0001	Yes	0.0004	Yes	< 0.0001	Yes	0.0018	Yes	< 0.0001
None 2 vs. ST 2	No	0.0638	Yes	0.0003	Yes	< 0.0001	Yes	0.0133	Yes	< 0.0001
PA 2 vs. PB 2	Yes	0.0105	Yes	< 0.0001	No	0.7044	No	0.068	Yes	< 0.0001
PA 2 vs. ST 2	Yes	0.0474	Yes	< 0.0001	No	0.708	Yes	0.0027	Yes	< 0.0001
PB 2 vs. ST 2	Yes	< 0.0001	No	0.9083	No	0.9961	Yes	0.004	No	0.2854

†, culture without PBMC
‡, culture with PBMC
