# Supplementary material for: Crosstalk between leukocytes triggers differential immune responses against Salmonella enterica serovars Typhi and Paratyphi
Source: PLoS Negl Trop Dis. 2019 Aug 14;13(8):e0007650. doi: 10.1371/journal.pntd.0007650 (PMC6709971; doi:10.1371/journal.pntd.0007650)
Supplement: Supplementary file 8 [file pntd.0007650.s004.docx]

S2 Table. Statistical analyses of **Fig 4C**

	IL-1β		IL-6		IL-8		TNF-α		CCL3	
1way ANOVA	Significant?	Individual *P* Value	Significant?	Individual *P* Value	Significant?	Individual *P* Value	Significant?	Individual *P* Value	Significant?	Individual *P* Value
None 1† vs. PA 1	Yes	0.0195	No	0.0541	Yes	0.0002	Yes	0.0264	Yes	0.0019
None 1 vs. PB 1	Yes	0.0255	Yes	0.0277	Yes	< 0.0001	Yes	0.0311	Yes	0.0155
None 1 vs. ST 1	Yes	0.0199	Yes	0.0278	Yes	0.0112	Yes	0.0257	Yes	0.0273
None 1 vs. None 2‡	No	0.1283	No	0.1024	No	0.9764	No	0.8546	No	0.2415
None 1 vs. PA 2	Yes	0.0007	Yes	0.0031	Yes	0.0008	Yes	0.0033	Yes	0.0058
None 1 vs. PB 2	Yes	0.0014	Yes	0.0058	Yes	< 0.0001	Yes	0.0007	Yes	0.0144
None 1 vs. ST 2	Yes	0.0173	Yes	0.0046	Yes	0.0002	Yes	0.0005	Yes	0.0289
PA 1 vs. PB 1	Yes	0.0488	No	0.1723	No	0.6061	No	0.3028	Yes	0.0184
PA 1 vs. ST 1	Yes	0.0262	No	0.1534	No	0.1369	Yes	0.0338	Yes	0.0067
PA 1 vs. None 2	Yes	0.0176	No	0.1392	Yes	0.0002	Yes	0.0264	Yes	0.0027
PA 1 vs. PA 2	No	0.5068	Yes	0.0151	No	0.6023	Yes	0.0284	Yes	0.0173
PA 1 vs. PB 2	Yes	< 0.0001	No	0.1904	Yes	0.0488	No	0.1629	No	0.9785
PA 1 vs. ST 2	No	0.2092	No	0.9622	No	0.9479	No	0.7771	No	0.5028
PB 1 vs. ST 1	Yes	0.0315	No	0.0571	Yes	0.0487	No	0.0736	No	0.1
PB 1 vs. None 2	Yes	0.0238	No	0.1835	Yes	< 0.0001	Yes	0.0308	Yes	0.0207
PB 1 vs. PA 2	No	0.4008	Yes	0.0002	No	0.3026	Yes	0.0011	Yes	0.0042
PB 1 vs. PB 2	No	0.1382	Yes	0.014	No	0.1373	Yes	0.0258	No	0.2906
PB 1 vs. ST 2	No	0.0573	No	0.0615	No	0.5615	No	0.2185	No	0.1878
ST 1 vs. None 2	Yes	0.017	No	0.2105	Yes	0.0104	Yes	0.0255	Yes	0.0327
ST 1 vs. PA 2	Yes	0.0078	Yes	0.0003	No	0.3259	Yes	0.0025	Yes	0.002
ST 1 vs. PB 2	Yes	< 0.0001	Yes	0.0135	Yes	0.0011	Yes	0.0071	No	0.0551
ST 1 vs. ST 2	No	0.0702	Yes	0.044	No	0.1542	Yes	0.0008	No	0.0932
None 2 vs. PA 2	Yes	0.0006	Yes	0.0142	Yes	0.0007	Yes	0.0032	Yes	0.0059
None 2 vs. PB 2	Yes	0.0012	Yes	0.0219	Yes	< 0.0001	Yes	0.0007	Yes	0.0129
None 2 vs. ST 2	Yes	0.0155	Yes	0.0248	Yes	0.0002	Yes	0.0005	Yes	0.0365
PA 2 vs. PB 2	Yes	0.0102	No	0.0934	Yes	0.0147	No	0.4159	Yes	0.0059
PA 2 vs. ST 2	No	0.0998	Yes	0.0279	No	0.6484	Yes	0.031	Yes	0.0031
PB 2 vs. ST 2	Yes	< 0.0001	Yes	0.0162	Yes	0.0424	Yes	0.046	No	0.7118

†, culture with Mφ-depleted PBMC

‡, culture with total PBMC
