# Supplementary material for: Crosstalk between leukocytes triggers differential immune responses against Salmonella enterica serovars Typhi and Paratyphi
Source: PLoS Negl Trop Dis. 2019 Aug 14;13(8):e0007650. doi: 10.1371/journal.pntd.0007650 (PMC6709971; doi:10.1371/journal.pntd.0007650)
Supplement: Supplementary file 9 [file pntd.0007650.s005.docx]

S3 Table. Statistical analyses of **S1 Fig**

	Trypan Blue		IL-6		IL-8		TNF-α		CCL3	
1way ANOVA	Significant?	Individual *P* Value	Significant?	Individual *P* Value	Significant?	Individual *P* Value	Significant?	Individual *P* Value	Significant?	Individual *P* Value
Media vs. PA 010020	No	0.4965	No	0.9975	Yes	< 0.0001	Yes	< 0.0001	No	0.3343
Media vs. PA CV223	No	0.5931	No	0.9785	Yes	< 0.0001	Yes	< 0.0001	No	> 0.9999
Media vs. PB 0203	No	0.0595	No	0.6805	Yes	< 0.0001	Yes	< 0.0001	No	0.0861
Media vs. PB CV23	No	0.0879	No	0.9995	Yes	< 0.0001	Yes	< 0.0001	No	0.9304
Media vs. ST Ty2	No	0.3124	No	0.9544	Yes	< 0.0001	Yes	< 0.0001	No	0.3343
Media vs. ST ISP1820	Yes	0.0475	Yes	0.0273	Yes	< 0.0001	Yes	< 0.0001	No	0.0635
Media vs. ST CV908	No	0.9963	No	0.9995	Yes	< 0.0001	Yes	< 0.0001	No	0.1157
PA 010020 vs. PA CV223	No	> 0.9999	No	> 0.9999	No	0.4725	No	0.1198	No	0.2024
PA 010020 vs. PB 0203	No	0.8686	No	0.3247	No	0.6308	No	0.125	No	0.9882
PA 010020 vs. PB CV23	No	0.9402	No	0.9373	No	0.1913	No	0.4537	No	0.9304
PA 010020 vs. ST Ty2	No	> 0.9999	No	0.6805	No	0.9521	No	0.9955	No	> 0.9999
PA 010020 vs. ST ISP1820	No	0.8144	Yes	0.0078	No	0.9985	No	0.9982	No	0.9678
PA 010020 vs. ST CV908	No	0.8709	No	0.9373	No	0.9996	No	0.9999	No	0.9969
PA CV223 vs. PB 0203	No	0.7925	No	0.2114	No	> 0.9999	No	> 0.9999	Yes	0.0466
PA CV223 vs. PB CV23	No	0.8881	No	0.8325	No	0.9977	No	0.984	No	0.797
PA CV223 vs. ST Ty2	No	0.9993	No	0.5122	No	0.9701	No	0.364	No	0.2024
PA CV223 vs. ST ISP1820	No	0.7277	Yes	0.0045	No	0.8107	No	0.3178	Yes	0.034
PA CV223 vs. ST CV908	No	0.9282	No	0.8325	No	0.7474	No	0.2377	No	0.0635
PB 0203 vs. PB CV23	No	> 0.9999	No	0.9166	No	0.9812	No	0.9863	No	0.5107
PB 0203 vs. ST Ty2	No	0.9698	No	0.9975	No	0.9952	No	0.3762	No	0.9882
PB 0203 vs. ST ISP1820	No	> 0.9999	No	0.4714	No	0.9198	No	0.329	No	> 0.9999
PB 0203 vs. ST CV908	No	0.1972	No	0.9166	No	0.8769	No	0.2468	No	> 0.9999
PB CV23 vs. ST Ty2	No	0.9923	No	0.9988	No	0.7325	No	0.8482	No	0.9304
PB CV23 vs. ST ISP1820	No	> 0.9999	No	0.0699	No	0.45	No	0.802	No	0.4178
PB CV23 vs. ST CV908	No	0.2748	No	> 0.9999	No	0.3854	No	0.696	No	0.609
ST Ty2 vs. ST ISP1820	No	0.9457	No	0.1885	No	0.9996	No	> 0.9999	No	0.9678
ST Ty2 vs. ST CV908	No	0.6902	No	0.9988	No	0.9982	No	> 0.9999	No	0.9969
ST ISP1820 vs. ST CV908	No	0.1618	No	0.0699	No	> 0.9999	No	> 0.9999	No	> 0.9999
